# Unraveling the determinants of microRNA mediated regulation using a massively parallel reporter assay

**DOI:** 10.1038/s41467-018-02980-z

**Published:** 2018-02-06

**Authors:** Ilya Vainberg Slutskin, Shira Weingarten-Gabbay, Ronit Nir, Adina Weinberger, Eran Segal

**Affiliations:** 1 Department of Computer Science and Applied Mathematics, Weizmann Institute of Science, 7610001 Rehovot, Israel; 2 Department of Molecular Cell Biology, Weizmann Institute of Science, 7610001 Rehovot, Israel

## Abstract

Despite extensive research, the sequence features affecting microRNA-mediated regulation are not well understood, limiting our ability to predict gene expression levels in both native and synthetic sequences. Here we employed a massively parallel reporter assay to investigate the effect of over 14,000 rationally designed 3′ UTR sequences on reporter construct repression. We found that multiple factors, including microRNA identity, hybridization energy, target accessibility, and target multiplicity, can be manipulated to achieve a predictable, up to 57-fold, change in protein repression. Moreover, we predict protein repression and RNA levels with high accuracy (*R* = 0.84 and *R* = 0.80, respectively) using only 3′ UTR sequence, as well as the effect of mutation in native 3′ UTRs on protein repression (*R* = 0.63). Taken together, our results elucidate the effect of different sequence features on miRNA-mediated regulation and demonstrate the predictability of their effect on gene expression with applications in regulatory genomics and synthetic biology.

## Abstract (web summary)

MiRNAs are known regulators of gene expression. Here the authors perform a large-scale massively parallel reporter assay to investigate the effect of a large number of designed 3′ UTR sequences on reporter expression and asses how miRNA regulatory elements features affect miRNA mediated repression.

## Introduction

Hundreds of miRNAs regulate the expression of about 60% of human protein-coding genes at the post-transcriptional level^1–5^. The mature miRNAs associate with Argonaute (AGO) proteins to form an miRNA-induced silencing complex (miRISC), which causes downregulation of target genes through a variety of mechanisms^6–9^. In animals, miRNA-specific binding sites, denoted as “seed matches”, are mostly localized within 3′ UTRs and usually contain six consecutive nucleotides, which are complementary to bases 2–7 of the 5′ end of the miRNA^1,3,6^. Although in some cases the seed match alone has been shown to confer regulation of target mRNAs^10^, in many cases additional sequence features were shown to affect regulation^11^. However, it is yet unclear to what extent these observations can be generalized in order to accurately predict the effect of miRNA regulatory elements (MREs) on gene expression.

A variety of methods have been introduced over the years that are aimed at studying the interaction between MREs and miRNAs. RNA immunoprecipitation-based methods reveal genome-wide physical interactions between the miRISC complex and the target mRNA, but they cannot elucidate which of these interactions have regulatory outcomes^12,13^. Quantification of gene expression after miRNA modulation can be used to identify enriched sequence features^4,11,14^. However, this approach is indirect, examines the gene expression response at non-native miRNA concentrations, and is often limited to the quantification of mRNA levels^15^. High-throughput proteomics and ribosome profiling following overexpression or knockout of miRNAs resolve most of the mentioned drawbacks, but they may still be affected by indirect and secondary effects^7–9,16^. Finally, reporter gene-based assays facilitate the testing of native and mutant 3′ UTR sequences in the presence and absence of miRNAs^10,17,18^. These approaches have limited throughput and are sensitive to the selection of the reporter construct and the miRNA concentration to resemble the native state^15^. New methods for the systematic analysis of the relationship between 3′ UTR sequence features and gene expression regulation are needed in order to further advance our understanding of the regulatory mechanisms involving MREs.

Recent advancements in high-throughput DNA synthesis facilitated the development of massively parallel reporter assays (MPRAs)^19–27^, allowing researchers to assess the regulatory consequences of thousands of variants in each experiment. Furthermore, systematic analysis of rationally designed libraries via MPRA has been previously shown to contribute to the understanding of a variety of regulatory mechanisms^19,20,25,28–30^. Despite previous 3′ UTR MPRAs for the discovery of multiple regulatory elements present in native sequences^31,32^, there is still a major gap in our ability to model the contribution of 3′ UTR sequence features to protein levels.

Here we set out to dissect the features governing MRE-mediated regulation of gene expression by applying an MPRA approach developed in our lab. We take advantage of the large scale of our assay and the rational design of the sequences to quantify the effect of MREs and surrounding sequences on protein repression for ten miRNAs. We find general and MRE-specific rules with protein repression reaching 57-fold. We integrate our findings into a comprehensive machine learning scheme achieving highly accurate predictions of protein repression and RNA levels from 3′ UTR sequence features alone. Finally, we accurately predict the difference in repression upon mutation of native MREs. Taken together, our models and analysis advance our understanding of MRE-mediated regulation and promote applications in regulatory genomics and synthetic biology.

## Results

### A massively parallel reporter assay for over 14,000 3′ UTRs

**Fig. 1:**
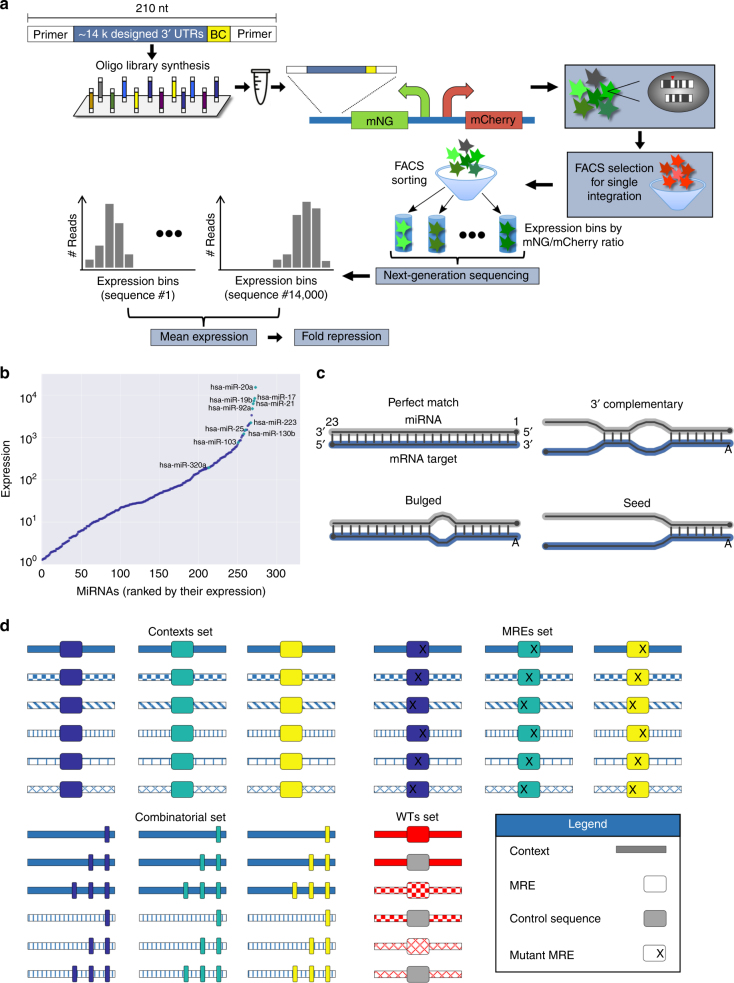
An experimental system for the systematic interrogation of the effects of variation in the 3′ UTR sequence on protein repression. **a** A schematic representation of our massively parallel reporter assay (Methods). **b** MiRNAs expressed in K562 cells as quantified by a published microarray experiment^34^. The ten highly expressed miRNAs selected for the library design are indicated. **c** An illustration of the four binding site types used for the design of MREs complementary to the ten selected miRNAs. MRE sequences were designed to match the depicted structure using ViennaRNA 2.0^60^. The perfect match MRE is a fully complementary binding site, disregarding the preference of the miRNA machinery for an “A” at the first position. For all other binding site types, the first position was forced to be an “A”. **d** An illustration of the four main mutagenesis schemes used in the design of the 3′ UTR sequences. First, we placed the designed MREs in a variety of native or designed contexts. Second, we subjected the MREs in the selected contexts to extensive mutagenesis of the binding sites. Third, we positioned multiple MREs in different architectures and compositions in selected contexts. Finally, we introduced mutations into native sequences that contained predicted MREs and included those in our library alongside the wild-type (WT) sequence. Different patterns depict different sequence contexts, while different colors depict different MREs. Designed mutations within the MRE sequence are annotated with an “X” and include single-base substitutions, single-base insertions, single- and double-base deletions, and cumulative mismatches from the MRE 5′ end. See also Supplementary Figure 1 and Methods

To get a quantitative measure for the effect of various sequence features on MRE activity, we adopted an MPRA approach previously used in our lab^20,33^. We designed 14,151 210 nucleotide (nt) long oligonucleotides, which are comprised of constant and variable regions (Methods). We applied systematic manipulations aimed to elucidate the consequence of specific sequence changes on miRNA-mediated regulation of gene expression. We generated a population of cells with a single variant per cell and quantified the mean expression and protein repression levels for each variant (Fig. 1a, Methods). We previously demonstrated that this approach is highly accurate and reproducible by obtaining high agreement (*R* = 0.98) between MPRA and isolated strains measurements of mean expression for a library of core promoter sequences^33^. Furthermore, we estimated the technical noise of our system by examining groups of ten variants with identical sequences except for the DNA barcode and found that the median relative standard deviation (RSD) was 10.5% (Supplementary Figure 1), indicating that our system exhibits low technical noise.

Here we designed synthetic MREs for ten miRNAs highly expressed in K562^34^ Fig. 1b). For each of these, we designed four MREs with varying base pair complementarity between the miRNA and the binding site (Fig. 1c). While the 8mer site maintains only marginally extended seed pairing, the 3′ complementary and bulged sites have additional 3′ pairing between the miRNA and the target, but they still lack the full complementarity required for cleavage of the target by the miRISC complex. This design allows us to test sequences that resemble native MREs as well as MRE types more relevant for synthetic biology, such as bulged and perfect match MREs, since they are essentially never observed in humans. We used these MREs, along with control sequences, in four major mutagenesis schemes (Fig. 1d).

**Fig. 2:**
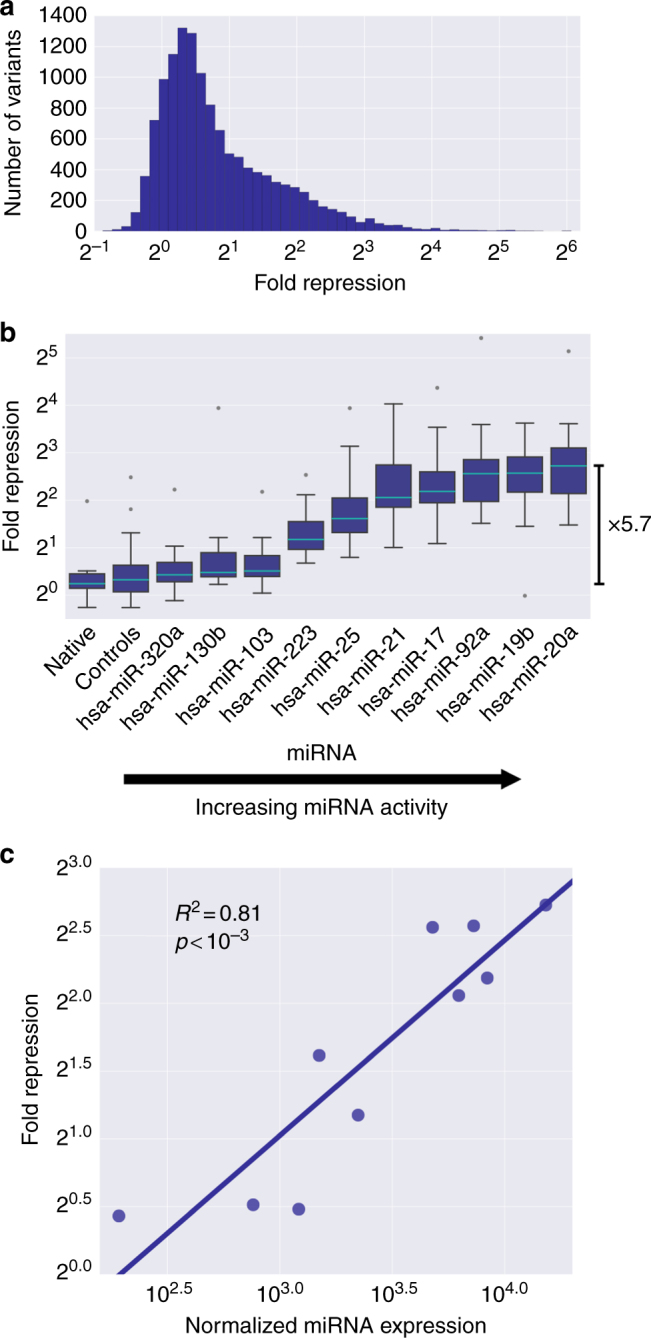
The miRNA abundance is a major determinant of the highly skewed protein repression distribution. **a** A histogram depicting the distribution of protein repression levels acquired by the method described in Fig. 1d. **b** Comparison of protein repression levels as a function of different miRNA identities. Each boxplot contains up to 20 variants generated by placing MREs for our ten selected miRNAs, perfectly matched to their miRNAs, in ten varying contexts at two positions, by replacing the existing sequence with the miRNA target site. The resulting data reveal that the position of the binding site had no significant effect (*p* = 0.96, Mann–Whitney *U* test). Therefore, we grouped the sequences by miRNA identity alone and examined its effect on repression. The bar depicts the 5.7-fold difference in median repression between hsa-miR-20a and the native contexts. **c** The median protein repression from **b** as a function of the miRNA expression levels, as quantified by a published microarray experiment^34^, for the ten selected miRNAs. Calculated *R*^2^ and associated *p*-values are indicated

We subjected the designed library to our experimental pipeline and obtained protein repression measurements for ~91.2% of the variants. We found that the assayed variants span over 50-fold in repression levels (Fig. 2a). The protein repression distribution is highly skewed, with the majority of sequences exhibiting little to no repression, consistent with the design of the library, which included a large proportion of MRE-destructive mutations. We conclude that our MPRA approach can measure the effect of 3′ UTR sequences on protein repression over a wide range of values.

### MiRNA abundance has a significant effect on repression

To investigate the effect of miRNA levels on their ability to repress their targets, we designed variants to robustly assess the miRNA activity using perfectly complementary MREs. The results show a range of repression levels, depending on the miRNA identity, that can span up to 5.7-fold in median repression (Fig. 2b). We computed the correlation between the median repression and the microarray data for the miRNA levels in K562^34^. We found that the miRNA abundance can explain up to 81% of the variability in the measured median repression (*R* = 0.90, *p* = 3.6e−04, Fig. 2c). Thus, these designed reporter constructs can serve as reliable sensors for miRNA expression. We confirm that miRNA abundance is a major determinant of target repression, at least for the subset of ten miRNAs included in our analysis.

### The MRE sequence is a major determinant of repression

**Fig. 3:**
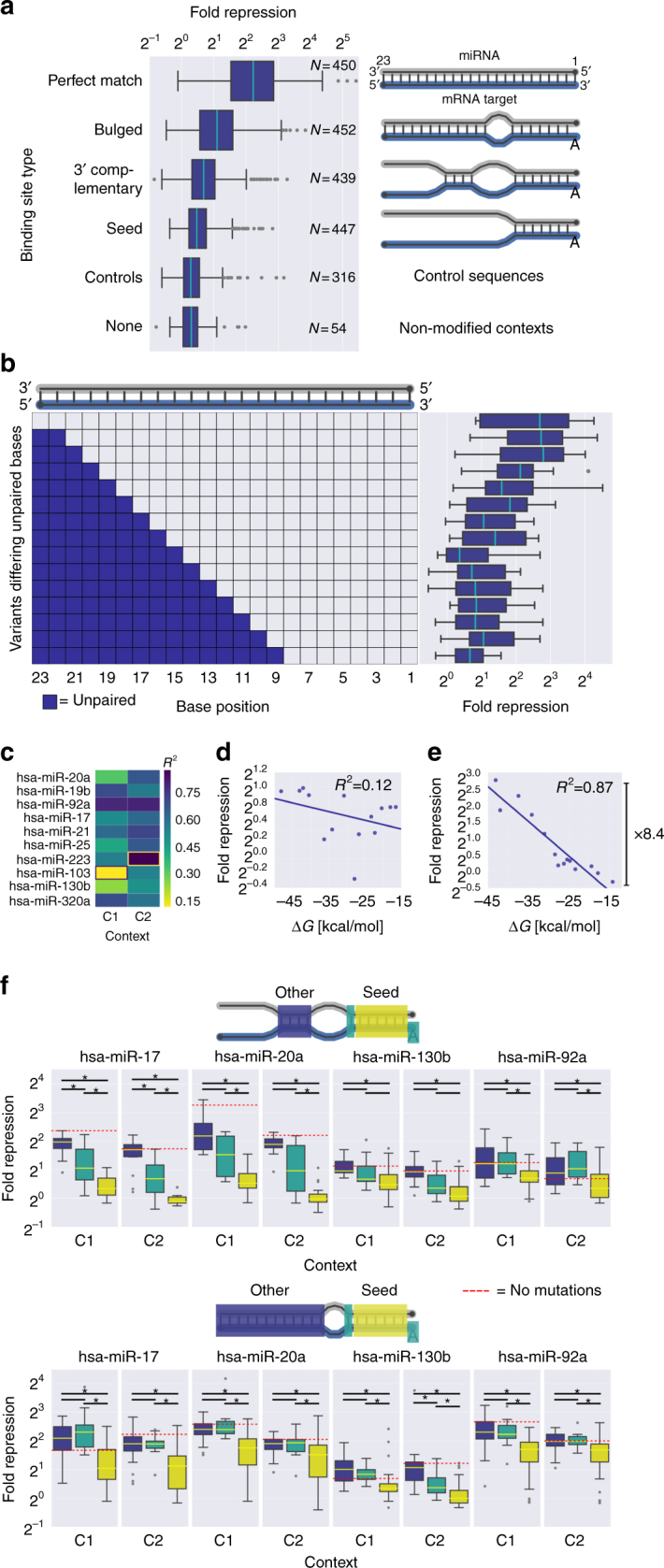
The MRE sequence and its interaction with the miRNA are a major determinant of repression efficiency. **a** Designed MREs of a given type for the selected ten miRNAs were placed in up to 57 contexts at a single copy in either of the two positions to generate a diverse set of variants. We grouped all the variants with the synthetic MREs by their binding site type, spanning different miRNAs and contexts, resulting in 2158 unique variants with measured repression. MRE types with a more extensive base pairing show higher repression. See also Supplementary Figure 2. **b** For each of the ten miRNAs, the perfectly complementary MRE was placed in one of the two contexts and mutated to accumulate mismatches from the MRE 5′ end. Each shaded square represents an unpaired base as predicted by ViennaRNA 2.0^60^. **c** A heatmap of the *R*^2^ values for the fold repression as a function of the hybridization energy (Δ*G*, calculated with RNAhybrid 2.1.2^68^) for each of the ten miRNAs in each of the two contexts. All of the correlations were negative. **d** Scatter plot of fold repression as a function of Δ*G* for hsa-miR-103 in context C1, highlighted in **c**. **e** Scatter plot of fold repression as a function of Δ*G* for hsa-miR-223 in context C2, highlighted in **c**. The bar annotates the 8.4-fold difference in fold repression spanned by the different mutants. **f** Comparison of the effect on fold repression of mutations in three regions of the MRE for four selected miRNAs for 3′ complementary (top panel) and bulged (bottom panel) MRE types. The first position is considered to be mutated if it is not an “A”. The effect of mutations in the different regions varies between miRNAs. Boxplot pairs marked with * are significantly different (*p* < 0.05, Mann–Whitney *U* one-sided test, FDR-corrected). The fold repression level of the non-mutated MRE in each context is indicated. In each variant, two identical copies of the MREs were placed in the context in order to increase sensitivity. See also Supplementary Figure 3

To study the effect of the MRE sequence on repression, we first examined the effect of the MRE type. The results reveal that MRE types with increased base pair complementarity between the miRNA and the MRE lead to stronger repression (Fig. 3a, Supplementary Figure 2). As expected, perfectly matched binding sites show the highest effect on repression, since they are the only examined binding site type that leads to cleavage by catalytically active AGOs and rapid RNA degradation^35–37^. Our results confirm on a larger scale that increased base pair complementarity correlates with a higher repression potential of MREs.

To further examine the effect of base pair complementarity, we took perfectly complementary MREs and accumulated mismatches between the miRNA and the MRE. We found that an increase in the number of mismatched bases led to progressively stronger reduction in repression, reaching saturation around base 14 of the miRNA (Fig. 3b). We further examined whether the decrease in repression can be explained by the hybridization energy (Δ*G*) between the miRNA and the MRE. Strikingly, we found that for most miRNAs, the coefficients of determination between the repression and the Δ*G* are high, indicating that between 12 and 87% of the repression can be explained by the Δ*G* (Fig. 3c–e). Moreover, the repression levels of the mutants can span up to 8.4-fold. Thus, these artificial MREs can be used for the design of 3′ UTRs with a predictable effect and serve as valuable synthetic biology parts. Together, these results confirm that the strength of the interaction between the MRE and the miRNA has a major effect on protein repression irrespective of miRNA identity.

Another important feature of the MRE sequence is the complementarity between the miRNA seed and the MRE. For the 3′ complementary and bulged sites, it is widely accepted that the mutations within the seed-complementary region are more destructive than the mutations in the other regions of the MRE^15^. Moreover, the importance of having an “A” at position 1 and/or base pairing at position 8 has been previously reviewed^3^. We examined these hypotheses by extensive mutational analysis of 3′ complementary and bulged MREs. Surprisingly, the results show that MREs differ in their sensitivities to mutations in the different regions (Fig. 3f and Supplementary Figure 3). Moreover, the effect size is different between the different MREs for both mutations in positions 2–7 and mutations in position 1 or 8. Thus, we propose that mutations within different regions of the MRE can have varied effects depending on the miRNA identity. Furthermore, we observe a difference in the effect size of reduction in repression upon mutation in the different groups when comparing between 3′ complementary and bulged MREs (e.g., hsa-miR-20a). Moreover, the reduction of repression in mutants with mutations at positions 2–7 becomes smaller. Although the bulged sites are rarely found in nature and represent an extreme case of 3′ compensation, this result teaches us about possible architectures of native MREs, where additional base pairs between the 3′ end of the miRNA and the MRE compensate for mutations within the seed region^2,38^. However, since we performed the mutational analysis on one 3′ complementary and one bulged site sequence for each miRNA, we cannot eliminate the possibility that the observed effect is sequence specific.

### Repression can be adjusted by modifying MRE flanking regions

**Fig. 4:**
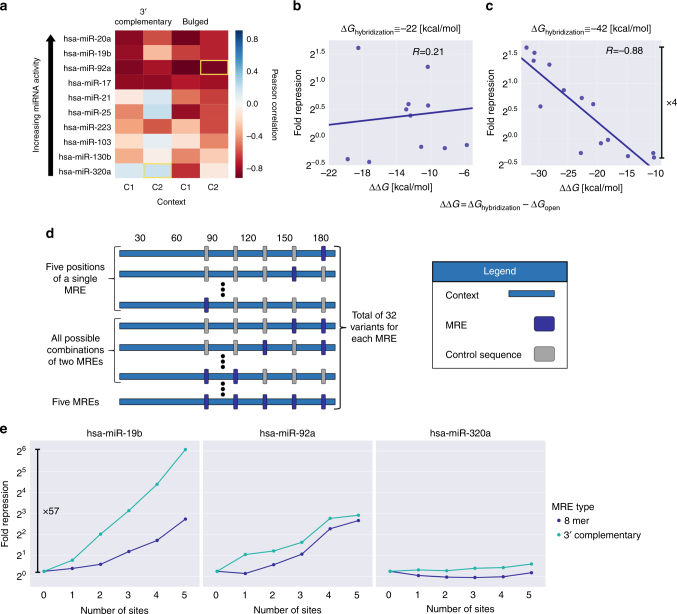
MRE accessibility and multiplicity can have a significant effect on fold repression. **a** For each of the ten selected miRNAs, either a 3′ complementary or a bulged MRE were placed in one of two contexts. The context was then mutated to vary the MRE accessibility by imposing different secondary structures using ViennaRNA 2.0^60^ predictions, resulting in 637 variants with measured repression. The ΔΔ*G* for each variant is computed as ΔΔ*G* = Δ*G*_hybridization_ – Δ*G*_open_, whereas Δ*G*_hybridization_ is the hybridization energy between the miRNA and the MRE and Δ*G*_open_ is the change in the ensemble free energy required to impose an unpaired MRE (Methods)^18^. The heatmap presents the Pearson correlation between fold repression and ΔΔ*G* for each MRE in each context. **b** Scatter plot of fold repression as a function of ΔΔ*G* for hsa-miR-320a in context C2, highlighted in **a**. **c** Scatter plot of fold repression as a function of ΔΔ*G* for hsa-miR-92a in context C2, highlighted in **a**. The bar annotates the 4-fold difference in fold repression spanned by the different mutants. **d** An illustration of the design approach for the MRE multiplicity analysis. Five positions were selected along the context. In each position either an MRE or a control sequence were placed, generating all 32 variants for a given MRE. This design was applied to a 3′ complementary and an 8mer MRE type for each miRNA, resulting in 570 unique variants with measured repression. **e** For each MRE, the variants were aggregated by the number of MRE sites and their median fold repression was plotted as a function of the number of sites. The bar annotates the 57-fold difference in fold repression that can be achieved by varying the MRE multiplicity for a 3′ complementary hsa-miR-19b MRE. See also Supplementary Figure 4

Although the MRE sequence, especially the seed region, has a major effect on repression, the RNA secondary structure surrounding it has been shown to significantly influence the interaction between the miRNA and the target sequence, thus affecting the extent of post-transcriptional regulation by the miRNA^18,39^. Therefore, we varied the sequence surrounding 3′ complementary and bulged MREs to affect the RNA structure encompassing them. As a measure of the total interaction energy, we use the ΔΔ*G* estimate as previously described^18^ (Methods). The results reveal that the variability in ΔΔ*G* can account for up to ~4-fold difference in repression with varied correlation values (Fig. 4a–c). Interestingly, we note that the MREs with a higher repression potential are associated with higher correlations. These results can be attributed to a smaller signal-to-noise ratio for the less potent sites or to miRNA and MRE type-specific effects. Thus, these insights help elucidate the sequence determinants required for higher MRE activity in both native and synthetic 3′ UTRs.

Repression has also been shown to increase with the number of MREs^10,11,40^. Therefore, we systematically varied the number of MREs for the selected miRNAs (Fig. 4d, Methods). We observe that for the more active miRNAs (hsa-miR-20a, hsa-miR-19b, and hsa-miR-92a) the fold repression increases as a function of the number of sites, up to a 57-fold difference in repression, while for the less active miRNAs (hsa-miR-21 and hsa-miR-320a), the fold repression remains low (Fig. 4e and Supplementary Figure 4). Furthermore, the increase in repression as a function of the number of MREs is qualitatively different for different miRNAs. For hsa-miR-92a, the repression potential reaches saturation at around four MREs, while for hsa-miR-20a and hsa-miR-19b this is not the case. These results indicate that an increase in the number of MREs can result in an increase in repression in an miRNA-specific manner. Given the artificial nature of this data set, it is most useful for the design of 3′ UTRs for synthetic biology purposes. However, the gained insight can still contribute to our understanding of native MRE clusters.

### Highly accurate prediction of repression from MRE features

**Fig. 5:**
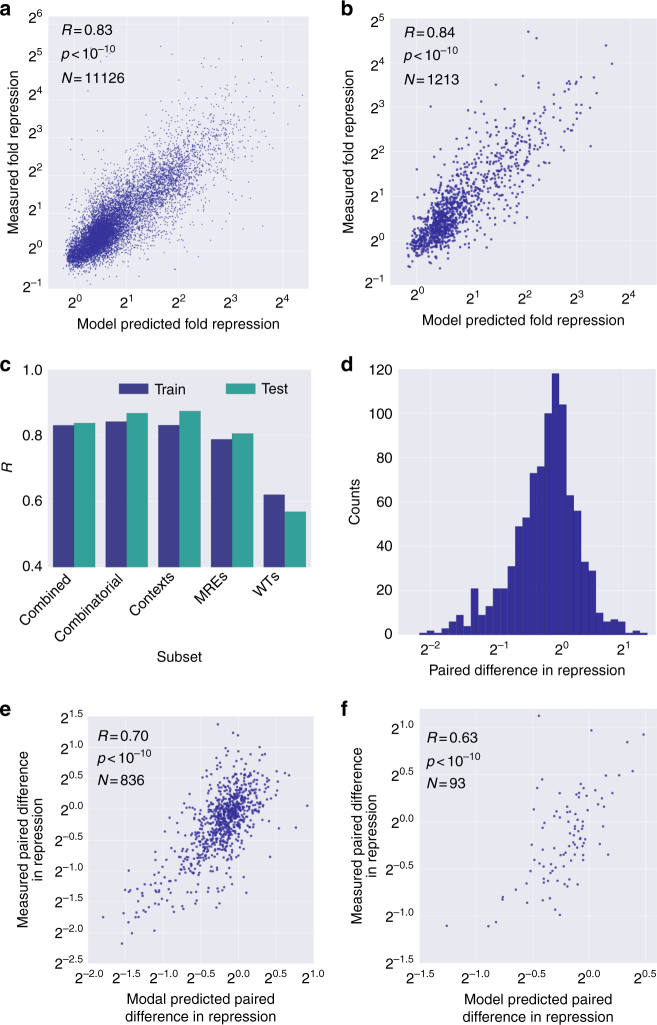
Highly accurate prediction of fold repression. **a** Scatter plot of measured vs model-predicted fold repression on the training data. To test model performance, we applied 10-fold cross validation (Methods). **b** Scatter plot of measured vs model-predicted fold repression on held out data. Pearson correlation was used as the measure of model performance. **c** Bar plot depicting model performance on training and test data. The model performance was assessed for each mutagenesis subset as in **a** and **b** for training and test sets, respectively. **d** A histogram depicting the distribution of paired difference in fold repression between a WT and mutant MRE. The data contain 929 WT and mutant pairs spanning 472 unique WT sequences. For each pair, the difference in fold repression was calculated by subtracting the fold repression of the WT from the fold repression of the mutant. Only 75 pairs spanning 45 WT sequences (~8%) show a difference greater than two-fold. **e** Scatter plot of measured vs model-predicted paired difference in fold repression on training data. The paired difference data were divided into training and test sets (10% of the data) and the MRE-based features for the WT and the mutant were concatenated resulting in 548 input features. We applied the same 10-fold cross validation approach as in **a** to generate model-predicted values and plotted them against the measured values. **f** Scatter plot of measured vs model-predicted paired difference in fold repression on held out data. We applied the same approach as in **b** to generate the predicted values and plotted them against the measured values

Based on our reported discoveries, we next ask whether we can build a comprehensive model for predicting repression from 3′ UTR sequence. A given 3′ UTR can interact with multiple miRNAs and have multiple MREs for a given miRNA. Therefore, the features in our model are a total interaction score for each of the 274 miRNAs detected in K562 cells^34^ (Methods). We apply a machine learning approach based on gradient boosting regression (GBR), which learns the contribution of each feature and can also learn non-linear interactions between the features^41^. We compare the measured to the predicted values from 10-fold cross validation (Fig. 5a) and used Pearson correlation as a measure of model performance. We repeated this analysis for each of our four mutagenesis schemes (Fig. 5c). Strikingly, our model predicts the observed expression in all of the synthetic sets with high accuracy (*R* = 0.79–0.84, *p* < 10^−10^) and also performs well in the more challenging task of mutations to WT MREs (*R* = 0.62, *p* < 10^−10^). This decrease in performance can be attributed to the features other than MREs contributing to the observed repression values in WT sequences or to a smaller signal-to-noise ratio given the smaller measured effect.

In our WT MRE mutagenesis set, we mutated predicted and validated MREs (Methods) by replacing the seed region or the entire MRE with an alternative sequence. By calculating the differences in repression between the native and the mutant sequences, we estimated the MRE repression efficiency. The distribution of the measurements reveals that most of the MRE mutations result in little or no effect on repression (Fig. 5d). Only ~8% result in more than a two-fold decrease in repression upon mutation of the MRE, supporting the notion that native MREs are used to fine tune gene expression. We adapted our machine learning pipeline to perform with two input 3′ UTRs and to predict the paired difference in repression (Methods). The predicted values show a surprisingly good agreement with the measured ones (*R* = 0.70, *p* < 10^−10^, Fig. 5e). These results indicate that mutations in MREs and surrounding sequences can affect gene expression levels in a highly predictable manner.

### RNA expression does not enhance predictive model accuracy

**Fig. 6:**
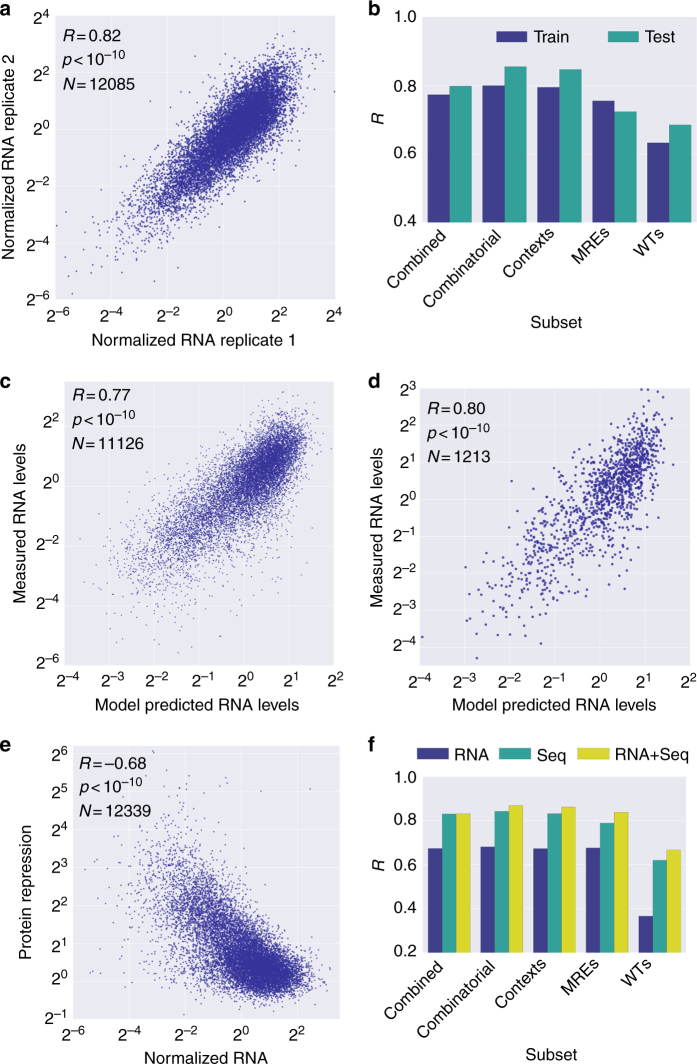
Incorporation of RNA levels into the model does not significantly improve model performance. **a** Scatter plot of RNA level measurement replicates from the genomically integrated reporter library. **b** Bar plot depicting model performance for predicting the RNA levels on training and test data. **c** Scatter plot of measured vs model-predicted normalized RNA levels on training data. **d** Scatter plot of measured vs model-predicted normalized RNA levels on test data. **e** Scatter plot of measured protein repression levels vs measured normalized RNA expression levels. **f** Comparison of model performances for predicting protein repression based on different training data. “RNA” refers to a model that used measured normalized RNA levels as the single input feature. “Seq” refers to the model that used the MRE-based features as in Fig. 5a. “RNA + Seq” refers to a model that used both the MRE-based features and the measured normalized RNA levels as input features

To gain further mechanistic insight into the regulatory process, we quantified RNA levels for our library. We adapted a previously used approach^24,42,43^ to estimate the RNA levels of each variant (Methods). We found that the method was highly reproducible between technical replicates (Fig. 6a, *R* = 0.82, *p* < 10^−10^). When applying our prediction pipeline to predicting the RNA levels from the MRE features, we achieved high performance, although slightly lower than the one achieved for prediction of protein repression (Fig. 6b, c). This may be due to the RNA data being more susceptible to technical noise as observed by higher median RSD (34%, Supplementary Figure 5A) and a wider distribution of RNA expression values (Supplementary Figure 5B).

With both the protein repression and RNA measurements at hand, we examined their joint distribution (Fig. 6e). We found a negative correlation (*R* = −0.68, *p* < 10^−10^) between protein repression and RNA abundance. We repeated our machine learning analysis and included the RNA abundance as the single input feature or in addition to the MRE-based features and compared the model performance (Fig. 6f). We found that using the RNA level as a single feature results in the lowest performance. Surprisingly, despite the correlation between RNA levels and protein repression, we found that the exclusion of the RNA levels from the feature set does not hinder model performance. Given that our model achieved high performance in predicting the RNA levels (*R* = 0.77, Fig. 6c), the model for predicting protein repression could capture the underlying RNA dynamics, resulting in high performance despite the exclusion of the RNA levels as a feature. These results indicate that RNA abundance is a consequence of sequence features and was captured well by our model.

### High performance of predictions on held out data

We further validated our model on a held out portion of our data that was not used at all during the model development. We processed the data and extracted the features in the same manner as we did in the main data set. Remarkably, our algorithm achieved similar performance on the held out data as it did on the main data set for predicting the repression in the different subsets for both the protein repression (*R* = 0.84, Fig. 5b) and RNA levels (*R* = 0.80, Fig. 6d). Furthermore, the algorithm for predicting the difference in repression between native and mutant MREs in WT sequences also achieved similar performance to the training set (*R* = 0.63, Fig. 5f). These results further support the ability of our algorithm to provide accurate predictions of MRE-mediated regulation in K562 cells.

### Features underlying repression and RNA level predictions

To investigate how different features contribute to prediction, we visualized the partial dependence (PD) of the model prediction on individual features using partial dependence plots (PDPs)^44^. PDPs graphically visualize the marginal effect of a given feature on prediction outcome after accounting for the average effect of all other features.

**Fig. 7:**
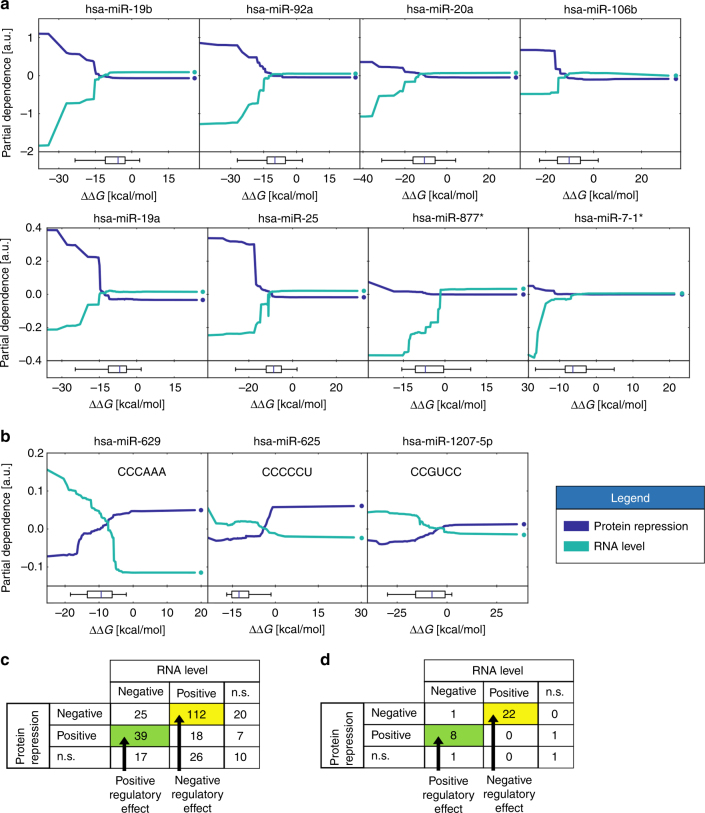
Features associated with negative and positive regulators underlying protein repression and RNA level predictions. **a** Partial dependence plots (PDPs) for selected features associated with a negative regulatory element depicting the contribution to the predicted protein repression and RNA level as a function of the ΔΔ*G*. For each feature, a stronger interaction between the miRNA and the MRE is reflected in a lower ΔΔ*G*. Boxplots (bottom) indicate the ΔΔ*G* percentiles (5, 25, 50, 75, and 95) of the distribution of the ΔΔ*G* values across all variants. Markers on the right end of each plot correspond to cases where the feature was not detected. Boxplots were calculated based on detected values only. **b** As in **a**, the PDPs for selected features corresponding to positive regulators. The hexamer sequence corresponding to each feature is indicated in each plot. **c** A table summarizing the number of features with a significant (FDR = 0.01) positive or negative Pearson correlation between the ΔΔ*G* and either protein repression or RNA levels. Positive and negative regulatory effects are defined in the text. n.s. non-significant. **d** As in **c** following filtering of the features for a minimal partial dependence range of 0.05 for both protein repression and RNA level

We calculated the PD for predictions of both protein repression and RNA levels from MRE features. For a feature associated with a negative regulatory element, we found lower ΔΔ*G* to correlate with lower RNA levels and higher protein repression levels (Fig. 7a). Similarly, for a feature associated with a positive regulatory element, we found lower ΔΔ*G* to correlate with higher RNA levels and lower protein repression (Fig. 7b). When neither of these correlations was significant, we classified the feature as contributing neither positively nor negatively. Overall, we found 112 features that correspond to the expected negative regulatory effect (Fig. 7c). Surprisingly, we also found 39 features corresponding to a positive regulatory effect. Since the magnitude of the PD contribution can vary greatly, we filtered the features and found 22 and 8 features corresponding to negative and positive regulatory effects, respectively (Fig. 7d). Of particular interest are the features that correspond to a positive regulatory effect (Fig. 7b). We found such examples only for features associated with lowly expressed or falsely annotated miRNAs. Therefore, these results may indicate that the rules learned by our model for these sequence features correspond to regulatory mechanisms other than miRNA-mediated regulation. Thus, this analysis identified the factors that underlie the high performance of our predictor and provided insights and testable hypotheses as to the regulatory mechanisms that drive miRNA-mediated repression.

A part of our high model performance could be attributed to the effect of miRNA expression levels on target repression. Therefore, we devised a basic model that would only consider miRNA expression levels and MRE multiplicity. We found that the performance for the basic model (*R* = 0.56) was lower than for our complete model (*R* = 0.84). To elucidate the contribution of additional factors to model performance, we chose to test a number of additional models (Methods). When comparing the model performance (Supplementary Figure 6), we found that our complete model achieves the highest performance. Judging by the performance of the simpler models, we can attribute the increase in performance to both the learned interactions between the features and the thermodynamic properties used in our complete model. Furthermore, we note the expected increase in performance whenever the number of MREs is considered as opposed to their occurrence alone. We conclude that despite the contribution of miRNA expression levels, our complete model still adds a substantial improvement to prediction.

### Reporter RNA levels are affected by varying miRNA profiles

To test the effect of varying miRNA profiles on expression we performed measurements of our library in four cell lines. Since the method for protein repression measurement was highly optimized for K562 cells, we modified our experimental approach to MPRA measurements of RNA from a transient transfection of the reporter library. This approach allowed us to perform the experiment in four selected cell lines: K562, MCF7, HEK293, and HepG2. We examined the miRNA microarray expression data for K562^34^, MCF7^45^, and HepG2^46^ to determine that the ranking of the miRNAs is different between these cell lines (Supplementary Figure 7). Notably, in MCF7 and HepG2, hsa-miR-21 is the most highly expressed miRNA as opposed to K562. Furthermore, hsa-miR-223 was not detected in HepG2, and was extremely lowly expressed in MCF7.

We performed transient transfection in the four cell lines and acquired normalized RNA level estimates for each variant (Methods). We found that the method is highly reproducible between technical replicates (Supplementary Figure 8A-D, *R* = 0.95–0.97, *p* < 10^−10^). Furthermore, for K562 cells, the measurements from transient transfection are in good agreement with the ones from genomic integration (Supplementary Figure 8E, *R* = 0.73, *p* < 10^−10^). Finally, we estimated the technical noise by estimating the median RSD, and found that our system exhibits low levels of technical noise (Supplementary Figure 8F). These values are lower than the 34% median RSD observed for the RNA measurements from the genomic integration experiment. We conclude that our transient MPRA method is a flexible approach for acquiring RNA measurements for reporter libraries in multiple cell lines.

**Fig. 8:**
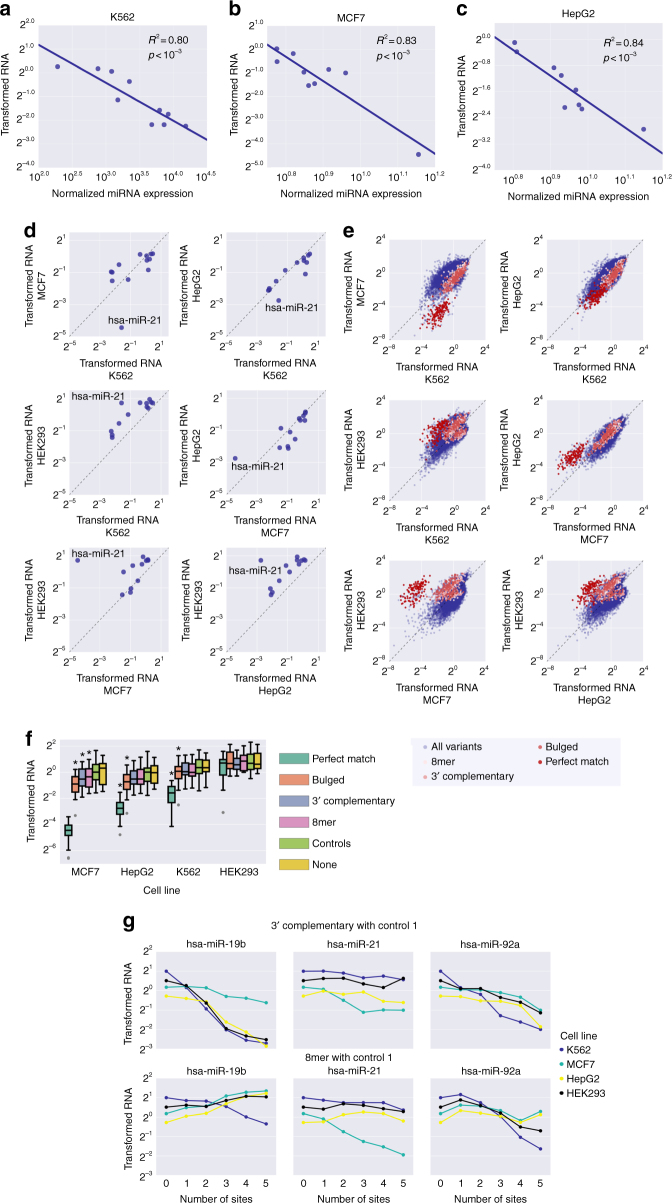
Varying miRNA profiles across cell lines affect reporter expression. **a**–**c** The median-transformed reporter RNA levels for perfect match MREs (see also Supplementary Figure 9) as a function of the miRNA expression levels, as quantified by published microarray for K562^34^, MCF7^45^, and HepG2^46^, for the ten selected miRNAs. Calculated *R*^2^ and associated *p*-values are indicated. For MCF7 and HepG2, hsa-miR-223 was excluded from this analysis due to its extremely low expression. **d** Pairwise comparison of transformed reporter RNA levels for perfect match MREs (see also Supplementary Figure 9) for the ten selected miRNAs. Hsa-miR-21 is annotated in every subplot. **e** Pairwise comparison of transformed RNA levels for all measured reporter library variants. The colored points indicate different MRE types for hsa-miR-21 as shown in the legend. **f** Transformed RNA levels of reporter constructs with different MRE types for hsa-miR-21 in different cell lines. Controls contain control sequences instead of the MRE. The "None" group contains the context with no inserted sequences. Boxplots marked with * are significantly different (*p* < 0.05, Mann–Whitney *U* one-sided test) from the controls group. See also Supplementary Fig. 10. **g** Transformed RNA levels as a function of number of MRE sites for K562, MCF7, HepG2, and HEK293 cells. Analysis was performed as in Fig. 4e per cell line. Plots shown are for 3′ complementary with control 1 and 8mer with control 1 at the top and bottom rows, respectively. Colors represent results from different cell lines as shown in the legend. A stronger effect is observed for hsa-miR-21 in MCF7 cells, where it has the strongest activity. See also Supplementary Fig. 11

We first examined the results for miRNA activity in all four cell lines, as we did in Fig. 2b (Supplementary Figure 9). To examine whether this difference in activity can be associated with miRNA abundance, we computed the correlation between the median expression and the microarray data for the miRNA expression in K562^34^, MCF7^45^, and HepG2^46^. We found that the miRNA abundance can explain up to 80%, 83%, and 84% of the variability in the measured median expression for perfectly complementary MREs in K562, MCF7, and HepG2 cells, respectively (Fig. 8a–c). Next we turned to comparing the miRNA activity between the cell lines (Fig. 8d). Clearly, hsa-miR-21 exhibits the most dramatic differences in activity in the pairwise comparisons. Thus, our assay captures the effect of miRNA levels on reporter expression in multiple cell lines and highlights the differences between them.

To further explore the differences in reporter expression between the cell lines, we plotted the pairwise comparisons for all the reporter constructs. Given the differences in hsa-miR-21 activity, we marked the variants with different hsa-miR-21 binding sites (Fig. 8e). It is clear that the most dramatic differences between the cell lines are dictated by the hsa-miR-21 MRE containing variants. Notably, the non-perfect match hsa-miR-21 MREs have a far smaller effect. Therefore, we subjected hsa-miR-21 to additional analysis, in which we directly compared the expression of variants with different hsa-miR-21 MREs in different contexts and across cell lines (Fig. 8f). We observe the expected effect of the MRE type in MCF7 cells, in which hsa-miR-21 has the highest activity. However, in cell lines where the hsa-miR-21 activity decreases, the effect of the weaker MRE types is diminished. We observe similar trends for the other miRNAs (Supplementary Figure 10). Thus, the effect of MREs for a given miRNA is dependent on both the miRNA activity and MRE sequence, across multiple cell lines.

Finally, we examined the effect of MRE multiplicity for five miRNAs, each with an 8mer or 3′ complementary MRE, across our four cell lines (Fig. 8g and Supplementary Figure 11). We observed that some MREs present varying trends between the cell lines, most notably hsa-miR-19b and hsa-miR-21. The accumulation of hsa-miR-21 MREs shows the largest effect in MCF7 cells, in which it is the most active miRNA. We conclude that the effect of MRE multiplicity depends on the MRE sequence and the miRNA expression profile determined by the cell line.

## Discussion

In this work, we systematically tested the quantitative effect of various MRE-related sequence features on reporter gene expression on an unprecedented scale using a MPRA for rationally designed 3′ UTRs. We used our measurements for ~13,000 3′ UTRs to dissect the sequence features governing MRE activity, and found general and miRNA-specific rules. We used the gained insight to develop a highly accurate model for predicting regulation from the 3′ UTR sequence on protein repression and RNA levels. Our algorithm achieves remarkable performance when predicting protein repression (*R* = 0.84), RNA levels (*R* = 0.80), and paired difference in protein repression upon mutation of native MREs (*R* = 0.63), providing a new model for the accurate prediction of the functional consequences of mutations within MREs. We examined the features underlying the predictions of our models and the identified expected and unexpected relationships between sequence features, protein repression, and RNA levels. Finally, we showed that the identified grammatical rules apply in multiple cell lines and respond to variations in the miRNA profile of the cells.

The MPRA system presented in this work is unique in multiple ways. First, our libraries are rationally designed, allowing for extensive testing of specific hypothesis by systematic mutagenesis of predefined features, as opposed to screening a limited number of native sequences with random mutations. Second, our MPRA is based on measurement of fluorescent reporters, which allows us to study regulation on both the RNA and protein level. Finally, we perform our measurements in a setting that closely resembles an unperturbed cellular environment, since our reporter gene is at a single copy in the genome and we do not manipulate the endogenous miRNA levels. Thus, our approach allows us to develop accurate models relevant to native scenarios.

Previous work performed mutational analysis of a limited number of 3′ UTR sequences interacting with a single or a very small number of miRNAs^10,17,18^. Furthermore, some of the analyzed features, such as MRE accessibility^18,39^ and MRE multiplicity^10,11,40^, were studied using multiple methods. Here we applied extensive mutagenesis on MREs for up to ten miRNAs, resulting in hundreds of mutants for each feature. This comprehensive approach allowed us to detect features that can be easily generalized to different miRNAs, such as the MRE type, while other features seem to be more dependent on the miRNA identity, such as MRE multiplicity. Furthermore, the quantitative nature of our measurements allows us to determine the impact of the examined features on repression. Such insights could not be achieved via lower throughput assays.

Many of our results can be informative regarding native binding sites and relevant for synthetic biology. These results include, but are not limited to, the effects of increased base pair complementarity, mutations with the MRE sequence, MRE accessibility, and MRE multiplicity. However, we acknowledge that mutagenesis of perfectly matched MREs, bulged MREs, or artificial cases of high MRE multiplicity are less reflective of native states and more applicable to synthetically designed 3′ UTRs. On the other hand, we note that only ~8% of the 929 pairs in our data set pairing WT and mutant MREs show a greater than two-fold difference in repression with a maximal value of 4.5-fold change in repression, this is in line with the previous reports^47^.

We note some limitations of our ability to rationally design systematic mutagenesis sets aimed to answer certain hypothesis. First, miRNAs hsa-miR-17 and hsa-miR-20a, and miRNAs hsa-miR-25 and hsa-miR-92a share the same seed, thus allowing cross interaction between the miRNAs and the MREs. Second, when introducing mutations to test a certain hypothesis, one cannot completely avoid changing other sequence and structure features. Since these design constraints are very hard to control for we can only accept them as part of the noise in our data.

Quantifying both RNA level and protein repression for the variants in our library allows us to examine the relationship between these two parameters (Fig. 6e). The Pearson correlation of −0.68 (*R*^2^ = 0.47) indicates that 47% of the variability in protein repression can be explained by RNA levels, in line with previous reports^48,49^. Interestingly, we note an absence of variants with high RNA abundance and high protein repression, supporting the idea that it might not be possible for miRNA-mediated regulation to repress translation without affecting RNA levels^8,9^.

Our model for predicting protein repression or RNA levels in K562 cells integrates the total ΔΔ*G* of each of 274 miRNAs. This approach takes into account the hybridization energy, MRE accessibility, and MRE multiplicity. These features have been previously used for the prediction of the effect of individual miRNAs^18,39^; however, to the best of our knowledge, this is the first work to incorporate these parameters for a large panel of miRNAs into a single predictor. This approach is beneficial for prediction, since MREs for different miRNAs can have interactions that can be captured by our GBR based model^11,40,41^.

Our algorithm predicts the level of repression for a given 3′ UTR as opposed to prediction of whether or not it is likely to be a target of a given miRNA. Thus, our algorithm can be used to predict, in K562 cells, whether a chosen 3′ UTR sequence will result in a specific desired expression level. A selection of 3′ UTRs can then be utilized to span the expression of genes in a regulatory or a metabolic pathway to study the effects of changes in protein expression or to optimize for a desired outcome. In addition, we present another algorithm that is capable of predicting the difference in repression upon mutation of native 3′ UTR sequences. This algorithm can serve as a tool for tuning the expression of an endogenous gene of interest in K562 cells to a desired level and study the resulting phenotypes. Thus, our algorithm can be utilized by the synthetic biology community to advance basic and applied research.

When analyzing the features underlying our predictor performance, we found a surprising number of features corresponding to positive regulatory elements (Fig. 7d). Since the ΔΔ*G* is calculated for each seed match (a hexamer sequence), it is possible that the hexamers used correspond to other regulatory sequences. Given that the miRNAs whose PDs are associated with a positive regulatory effect are relatively lowly expressed or falsely annotated, it is possible that the function learned by our predictor is associated with a different regulatory mechanism. The hexamer sequences can be binding sites for RNA binding proteins (RBPs) that are capable of stabilizing the mRNA or increasing translation efficiency. For example, the hexamer “CCCCCU” corresponds to a known poly-C motif, which is bound by a family of RBPs and was shown to contribute to RNA stabilization and translation enhancement^50,51^. Future endeavors may devise a model that will include features of regulatory elements other than MREs, thus improving our ability to predict the protein repression and mRNA expression from sequence alone.

Finally, we examined the effect of varying miRNA profiles on reporter expression by measuring RNA levels for our library in four different cell lines. The presented data greatly expand the accumulated evidence for the sequence features affecting MRE activity in human cells. The results show that the miRNA levels are a major determinant of MRE activity and reveal that the most prominent differences in reporter expression between the cell lines can be attributed to MREs of hsa-miR-21, whose expression differs the most between the cell lines. We corroborate the effect of main MRE and surrounding sequence features from K562 cells in the newly tested cell lines, indicating that the devised rules can be generalized.

In summary, we used a quantitative high-throughput assay to measure the regulatory effect of over 14,000 rationally designed 3′ UTRs to decipher the rules of miRNA-based regulation. We identified various general and miRNA-specific sequence features responsible for the potential to repress translation. Furthermore, we combined our insights into accurate models for prediction of protein repression and RNA levels. These results contributed to a systematic functional understanding of miRNA-mediated gene regulation, as well as opened new possibilities for making use of the powerful regulatory potential of this mechanism for synthetic biology.

## Methods

### Synthetic library design

General design notes: oligonucleotides were designed to maintain a constant length of 210 nt. We made sure that all sequences excluded restriction sites used for cloning and the canonical polyadenylation signal (AATAAA) and its point mutants. All the variants were composed of an 18 nt forward primer, 12 nt barcode sequence, 162 nt variable region, and 18 nt reverse primer sequences. The 14,151 variants were designed as a part of a larger library of 55,000 variants. Unique primer sequences were used to facilitate targeted amplification of the 14,151 variants pool.

Selection of miRNAs and contexts: the miRNAs were selected based on a literature survey and miRNA expression data as quantified by microarray^34^. We selected miRNAs that were highly expressed (within the top 10%, except for hsa-miR-320a). We selected miRNAs involved in different cell processes such as cancer (hsa-miR-17-92 cluster^52,53^ and hsa-miR-21^53–55^) and K562 differentiation (hsa-miR-223^56^, hsa-miR-103^57^, and hsa-miR-320a^57,58^). The selected miRNAs were as follows: hsa-miR-20a (MIMAT0000075), hsa-miR-17 (MIMAT0000070), hsa-miR-19b (MIMAT0000074), hsa-miR-21 (MIMAT0000076), hsa-miR-92a (MIMAT0000092), hsa-miR-223 (MIMAT0000280), hsa-miR-25 (MIMAT0000081), hsa-miR-130b (MIMAT0000691), hsa-miR-103 (MIMAT0000101), and hsa-miR-320a (MIMAT0000510). Underlined miRNAs were subject to more extensive mutagenesis in some cases.

To facilitate library design and to examine the effect of varying native contexts, we selected 57 unique sequences. The sequences spanned a range of GC content and minimal folding energy (MFE) values. Origins of the sequences were coding regions, 5′ UTRs, 3′ UTRs, viral genomes, and random sequences.

Design of the synthetic MREs and controls: for each miRNA, the target sites were generated based on the miRNA sequence from miRBase version 20^59^. We designed four synthetic MREs for each miRNA: perfectly matched, bulged, 3′ complementary, and seed-matched. For perfectly matched MREs, we took the reverse complementary sequence to the miRNA sequence. For bulged sites, we enforced a bulge at bases 9–11 of the miRNA. For 3′ complementary sites, we enforced a perfect match within the seed region and additional base pairing at bases 13–16 of the miRNA, while enforcing the rest of the MRE to be unpaired. For seed-matched sites, we enforced pairing within the seed region alone, while enforcing the rest of the MRE to be unpaired. Secondary structure predictions were performed with ViennaRNA package 2.0^60^. All target sites were forced to be 23 nt long (for shorter miRNAs additional bases from the pre-miRNA were taken) except for bulged sites, which were 22 nt (due to deletion in the target site required to form the bulge). Three 23 nt long control sequences were used throughout the library and included a CXCR4 siRNA perfect match site^17^, a 23 nt sequence from firefly Luciferase, and a random 23 nt sequence.

Designing the contexts set: we placed MREs in native contexts by replacing the native sequence in the selected positions, thus maintaining constant oligonucleotide length. We placed MREs for the selected three miRNAs in 50 different contexts. We also placed MREs for all the miRNAs in a subset of ten contexts. The MREs were placed in two positions within the oligonucleotide. In addition, we placed the MREs for the three selected miRNAs in two selected contexts in 14 different positions with 10 nt difference between them. For a more focused approach in examining the effect of MRE accessibility, we placed 3′ complementary and bulged MREs for all the ten miRNAs in various predicted MFE structures (ViennaRNA package 2.0^60^).

Designing the mutant MREs set: two adjacent (1 nt distance between upstream site 3′ end and downstream site 5′ end) mutant target sites, in order to increase sensitivity, were placed in two selected contexts. The generated mutations included cumulative mismatches in perfect and bulged MREs (mismatches were predicted to be unpaired with ViennaRNA package 2.0^60^), all point mutants of the seed in bulged MREs, single-base pair mutations and double-base pair mutations to complement bases, single-base deletions, “C” and “G” insertions in bulged, and 3′ complementary MREs. In addition, for all the MREs, except the perfect match ones, mutants with a first position (from miRNA 5′ end) “A”, match or mismatch, were included. The seed region was defined as the MRE bases complementary to bases 2–7 of the miRNA.

Designing the combinatorial set: to examine the effect of MRE multiplicity, we selected five positions along the oligonucleotide sequence. In each position we placed either an MRE or a control sequence, generating all 32 (2^5^) variants for a given MRE and resulting in identical sites with different multiplicity. This design was applied to a 3′ complementary and an 8mer MRE for each miRNA and was repeated for a total of two control sequences. We also placed various pairwise combinations of MREs in multiple context sequences and at varying distances between the MREs. Since these designs did not yield significant results and were only used in the prediction pipeline, their description is beyond the scope of this work.

Designing the WTs set: we collected native sequences containing predicted and verified MREs from a number of sources. First, we compiled a list of 114 sequences of verified targets of our ten miRNAs based on public databases^61,62^. We mutated these targets to span various lengths of the native region, all including the MRE, in two different context sequences (to maintain constant oligonucleotide length). We also replaced the native MRE with our correspondingly designed MREs (interacting with the same miRNA) and control sequences. Second, we extracted native sequences with MREs for our ten miRNAs from a database of informative single-nucleotide polymorphisms (SNPs) in close proximity of miRNA-binding sites and mutated the seed region for each reported allele. Finally, we included native sequences with predicted MREs by selected prediction tools: TargetScan6.2^11^, PICTAR2.0^63^, and PITA^18^.

Finally, a large number of WT sequences was incorporated into the library and mutated based on target site prediction, verified target sites^61,62^ and informative SNPs in close proximity of miRNA-binding sites^64^. For each of our ten miRNAs, the top 100 sites based on a score assigned by the program, were selected. In addition, the predicted sites of all three programs for our ten miRNAs were intersected, and the common list of 134 sites was added to the list of target sites. For each target site, two mutants were generated by replacing the seed with a control octamer.

Overall, of the 472 unique WT sequences used for the various mutagenesis schemes, 201 contained 8mer sites, 215 contained 7mer-m8 sites, 44 contained 7mer-A1 sites, and 12 contained 6mer sites, which were described elsewhere^3^. The selected MREs were under selective pressure as determined by a higher conservation score of the MREs vs the rest of the native sequence in our oligos (*p* < 10^−10^, Wilcoxon signed-rank test, Supplementary Figure 12).

Designing a set of variants with multiple barcodes: we selected 20 variants expected to span a large range of repression levels. For each variant, we generated ten different barcodes. Only one of the barcoded variants was randomly selected for all other downstream analysis.

### Experimental procedures

Cell culture: K562 cells were acquired from ATCC. The cells were grown in Iscove’s Modified Dulbecco's Medium supplemented with 10% fetal bovine serum (Biological Industries) and 1% Penicillin–Streptomycin solution (Biological Industries). The cells were split when reaching a concentration of ~10^6^ cells/ml. The cells were grown in an incubator at 37 °C and 5% CO_2_. The cells were frozen in batches of 4 × 10^6^ cells in growth medium supplemented with 5% DMSO.

MCF7, HepG2, and HEK293 cells were kindly contributed by Professor Moshe Oren and were grown in Dulbecco’s Modified Eagle’s Medium supplemented with 10% fetal bovine serum (Biological Industries) and 1% Penicillin–Streptomycin solution (Biological Industries). The cells were maintained at 30–90% confluency. The cells were grown in an incubator at 37 °C and 5% CO_2_. The cells were frozen in batches of 4 × 10^6^ cells in growth medium supplemented with 10% DMSO.

The cell lines were tested for mycoplasma contamination using EZ-PCR Mycoplasma Kit (Biological Industries).

Construction of the master plasmid: the desired full construct was assembled using golden gate assembly in the pFus_A vector (kindly provided by J. Hanna, Weizmann Institute of Science). The target construct was split into five parts based on the available templates (previously generated in our lab) and desired additional features (such as restriction sites) to be inserted between parts. The *mNeonGreen* sequence was licensed from Allele Biotechnology, San Diego, CA. Assembly overhangs were chosen to be compatible with the destination vector (pFus_A), have at least two mutations difference between them, and a GC content of 25%. Primers were designed to include the BsaI site with additional bases at the most 5′ end of the primer followed by the relevant overhang, additional features (for example a restriction site), and complementary region to the relevant template.

The five parts were PCR-amplified using a KOD hotstart polymerase (Merck Millipore) according to the manufacturer’s protocol with annealing temperature of 55°C and elongation time of 2 min (determined by the longest product) for 35 cycles. The products were gel-purified from a 1% agarose gel stained with GelStar (Cambrex Bio Science Rockland) using a gel extraction kit (Qiagen). The purified products were used in a golden gate assembly reaction, which typically included 150 ng of destination vector (pFus_A), equimolar amount of each of the five parts, 2 μl 10× T4 ligase buffer (NEB), 2 μl BSA 10×, 1 μl BsaI enzyme (NEB), 1 μl quick ligase (NEB), and water to a final volume of 20 μl. The reaction was incubated for 12 cycles of 37 °C for 5 min (restriction) and 16 °C for 10 min (ligation), followed by 10 min at 80 °C (heat inactivation). Since one of the fragments contained an internal BsaI site, 1 μl of quick ligase was added and the reaction was incubated at 20 °C for 30 min for final ligation and 65 °C for 10 min (heat inactivation). The products were kept at 4 °C until transformation. The reaction products were transformed into chemically competent HIT-DH5α *Escherichia coli (E. coli)* (RBC Bioscience) using a standard heat shock transformation protocol. Typically 8 μl of reaction products were transformed into 100 μl of cells and plated on LB plates supplemented with Spectinomycin (50 μg/ml final concentration), IPTG (100 μl of 0.1 M solution spread on the plate), and Xgal (40 μl of 40 mg/ml solution spread on the plate). One positive clone (verified via Sanger sequencing) was digested with XbaI and PmeI (NEB), and the insert was cloned into pZDonor (CompoZr® Targeted Integration Kit - AAVS1 kit, SIGMA) digested with XbaI and PmeI, using quick ligase (NEB) and transformed into DH5α *E. coli* (RBC Bioscience) using a standard heat shock transformation protocol. The transformed cells were selected on LB+Amp plates (100 μg/ml final concentration). Correct clones were verified using Sanger sequencing.

Synthetic library production and amplification: the production and amplification steps were adopted from a protocol that was previously described for yeast promoters^19^. We used Agilent oligo library synthesis technology to produce a pool of 55,000 different fully designed single-stranded 210-oligomers (Agilent Technologies, Santa Clara, CA), a subset of which comprised the 14,151 pool, used in this study, defined by unique amplification primers. Each designed oligo contains common priming sites and restriction sites, leaving 174 for the variable region. The library was synthesized using Agilent’s on-array synthesis technology^65,66^, and then provided to us as an oligo pool in a single tube (10 pmol). The pool of oligos was dissolved in 200 μl Tris-ethylenediaminetetraacetic acid (Tris-EDTA), and then diluted 1:50 with Tris-EDTA, which was used as template for PCR. We amplified the library using PCR in 16 tubes. Each PCR reaction contained 19 μl of water, 5 μl of DNA, 10 μl of 5 × Herculase II reaction buffer, 5 μl of 2.5 mM deoxynucleotide triphosphate (dNTPs) each, 5 μl of 10 μM forward (Fw) primer, 5 μl of 10 μM reverse (Rv) primer, and 1 μl Herculase II fusion DNA polymerase (Agilent Technologies). The parameters for PCR were 95 °C for 1 min, 14 cycles of 95 °C for 20 s, and 68 °C for 1 min, each, and finally one cycle of 68 °C for 4 min. The oligonucleotides were amplified using constant primers in the length of 35 nt, which have 18 nt complementary sequence to the single-stranded 210-mers and a tail of 17 nt containing SpeI (Fw primer) and AscI (Rv primer) restriction sites. The primer sequences for the 14,151 pool, used in this study, are as follows, underline represents the 18 nt complementary sequence to the ssOligos: AAGTTCAACTAGTACGTCGAAATGGGCCGCATTGC (Fw primer) and CCCTTGGCGCGCCTCTCTCGTCATCAGCCGCAGTG (Rv primer). The PCR products from all 16 tubes were joined and concentrated using Amicon Ultra, 0.5 ml 30 K centrifugal filters (Merck Millipore) for DNA purification and concentration. The concentrated DNA was then purified using a PCR mini-elute purification kit (Qiagen) according to the manufacturer’s instructions.

Synthetic library cloning into reporter master plasmid: library cloning into the master plasmid was adopted from a protocol that was previously described for yeast promoters^19^. Purified library DNA (540 ng total) was cut with the unique restriction enzymes SpeI and AscI (Fermentas FastDigest) for 2 h at 37 °C in three 40 μl reactions containing 4 μl fast digest (FD) buffer, 1 μl of SpeI enzyme, 1 μl of AscI enzyme, 18 μl of DNA, and 16 μl of water, followed by a heat inactivation step of 20 min at 65 °C. Digested DNA was separated from smaller fragments and uncut PCR products by electrophoresis on a 2.5% agarose gel stained with GelStar (Cambrex Bio Science Rockland). Fragments the size of ~221 nt were cut from the gel and eluted using electroelution Midi GeBAflex tubes (GeBA, Kfar Hanagid, Israel). The eluted DNA was precipitated using sodium acetate–isopropanol. The master plasmids were cut with SpeI and AscI (Fermentas FastDigest) in a reaction mixture containing 6 μl of FD buffer, 3 μl of each enzyme, and 3.55 μg of the plasmid in a total volume of 60 μl. After incubation for 2 h at 37 °C, 3 μl of FD buffer, 3 μl of alkaline phosphatase (Fermentas), and 24 μl of water were added and the reactions were incubated for an additional 30 min at 37 °C followed by a heat inactivation step of 20 min at 65 °C. The digested DNA was purified using a PCR purification kit (Qiagen). The digested plasmids and DNA library were ligated for 0.5 h at room temperature in two 10 μl reactions, each containing 150 ng plasmid and the library in a molar ratio of 1:1, 1 μl CloneDirect 10× ligation buffer, and 1 μl CloneSmart DNA ligase (Lucigen Corporation), followed by a heat inactivation step of 15 min at 70 °C. Ligated DNA (14 μl) was transformed into a tube of *E. coli* 10 G electrocompetent cells (Lucigen) divided into seven aliquots (25 µl each), which were then plated on 28 Luria broth (LB) agar (200 mg/ml amp) 15-cm plates. To ensure that all 14,151 oligos are represented, we collected ~0.5 × 10^6^ colonies 16 h after transformation, by scraping the plates into LB medium. Library-pooled plasmids were purified using a NucleoBond Xtra maxi kit (Macherey Nagel). To eliminate possible leftovers of insert DNA, which compromises nucleofection efficiency, we treated the library-pooled plasmids with Plasmid-Safe™ ATP-Dependent DNase (Epicentre) by mixing 20 µl of library-pooled plasmids (7.5 µg), 5 μl 10 × buffer, 5 μl 10 mM ATP, 1 μl Plasmid-Safe™ ATP-Dependent DNase, and 19 μl water and incubating at 37°C for 40 min, followed by heat inactivation at 70°C for 30 min. Finally, we purified the mixture by standard sodium acetate–ethanol precipitation.

To ensure that the collected plasmids represent a ligation of single inserts, we performed colony PCR on 96 random colonies. The volume of each PCR reaction was 30 μl; each reaction contained a random colony picked from an LB plate, 3 μl of 10 × DreamTaq (Thermo Fisher Scientific) buffer, 0.6 μl 10 mM dNTPs mix, 0.9 μl 25 mM MgSO_4_, 1.2 μl of 10 μM 5′ primer, 1.2 μl of 10 μM 3′ primer, 0.3 μl DreamTaq polymerase, and 22.8 μl water. The parameters for PCR were 95 °C for 2 min, 35 cycles of 95 °C for 30 s, 55 °C for 30 s, and 72 °C for 50 s, each, and finally one cycle of 72 °C for 5 min. The primers used for this reaction were CACTCCAAGACCGAGCTCAACTTC (Fw primer) and GGGGCTTTTCTGTCACCAATCC (Rv primer). Of the 96 colonies tested, only four had multiple inserts.

Transfection into K562 cells and genomic integration: the purified plasmid library was transfected into K562 cells and genomically integrated using the Zinc Finger Nuclease (ZFN) system for site-specific integration, using the CompoZr® Targeted Integration Kit - AAVS1 kit (SIGMA). Transfections were carried out using Amaxa® Cell Line Nucleofector® Kit V (LONZA). To ensure library representation, we carried out 12 transfections of the purified plasmid library. A master plasmid with no insert was also genomically integrated in the same manner. For each transfection, 4 × 10^6^ cells were centrifuged and washed twice with 20 ml of Hank’s Balanced Salt Solution (HBSS, SIGMA). The cells were resuspended in 100 μl solution (warmed to room temperature) composed of 82 μl solution V and 18 μl supplement (Amaxa® Cell Line Nucleofector® Kit V). Next, the cells were mixed with 2.75 μg of donor plasmid and 0.6 μg ZFN mRNA (prepared in-house) just prior to transfection. Transfection was carried out using program T-16 on the Nucleofector^TM^ device, immediately mixed with ~0.5 ml of pre-cultured growth medium and transferred to a six-well plate with additional 1.5 ml of pre-cultured growth medium. A purified plasmid library was also transfected without the addition of ZFN and served as a control to determine when the non-integrated plasmid signal was diminished. Non-transfected cells (1.5 × 10^6^) were taken after the washes in HBSS and seeded in 2 ml of pre-cultured growth medium, serving as an additional control when sorting the cells in the FACS.

Transient transfection of K562 cells was performed using Lipofectamine® 2000 (ThermoFisher Scientific) following the manufacturer protocol. The day of the transfection, 5 × 10^6^ cells were plated in 10 ml growth media without antibiotics and transfected using 20 μg of donor plasmid and 50 μl of Lipofectamine® 2000. After 4 h, the cells were centrifuged and resuspended in 20 ml complete growth media. The cells were harvested for RNA purification 24 h after transfection. The transfections were performed in two replicates.

Transient transfection of MCF7, HepG2, and HEK293 cells: MCF7, HepG2, and HEK293 cells were plated in 10 cm plates 24 h before transfection making sure they are at 70–90% confluency on the day of transfection. The cells were transfected with X-tremeGENE HP (Sigma) according to the manufacturer protocol. A ratio of 3:1 X-tremeGENE HP:DNA was used with 10 μg of donor plasmid. The cells were harvested for RNA purification 24 h after transfection. Transfections were performed in two replicates.

Sorting the library by FACS: K562 cells were grown for 22 days to ensure that non-integrated plasmid DNA was eliminated, confirmed by the cell line transfected without ZFNs. A day prior to sorting, the cells were split to ~0.3 × 10^6^ cells/ml. On the day of sorting, the cells were centrifuged, resuspended in sterile PBS, and filtered using cell-strainer-capped tubes (Becton Dickinson (BD) Falcon). Sorting was performed with BD FACSAria II SORP (special-order research product) at low sample flow rate and a sorting speed of ~13000 cells per second. To sort cells that integrated the reporter construct successfully and in a single copy (~4% of the population), we determined a gate according to mCherry fluorescence so that only mCherry-expressing cells corresponding to a single copy of the construct were sorted (mCherry single integration population). We collected a total of ~6.3 × 10^6^ cells in order to ensure adequate library representation. We also sorted the master plasmid nucleofected cells for single-copy integration by collecting 0.15 × 10^6^ cells. The cells were grown for 13 days before the next sorting step, during which batches of 4 × 10^6^ cells were frozen for future experiments. To obtain high-resolution measurements of mNeonGreen levels, we sorted the mCherry single integration population into 16 bins according to the mNeonGreen/mCherry ratio. Each bin was defined to span a range of mNeonGreen/mCherry ratio values, such that it contains between 1 and 10% of the mCherry single integration population cells and maintains high sorting efficiency. We collected a total of 1.55 × 10^7^ cells in order to ensure adequate library representation. The cells from each bin were grown for freezing and purification of genomic DNA. In addition, the sorted master plasmid nucleofected cells were subjected to analysis in order to determine the distribution of mNeonGreen/mCherry ratios for the vector with no library insert.

Genomic DNA and RNA purifications: for each of the 16 bins, we purified genomic DNA by centrifuging 5 × 10^6^ cells, washing them with 1 ml PBS and purifying DNA using DNeasy Blood & Tissue Kit (Qiagen), according to the manufacturer's protocol. In addition, we purified genomic DNA from the mCherry single integration population by centrifuging 16 × 10^6^ cells, washing them with 4 ml PBS, splitting into four tubes of 1 ml, and purifying DNA using DNeasy Blood & Tissue Kit (Qiagen) according to the manufacturer's protocol. This purification was performed in two replicates.

For the cell population sorted for single integration of the reporter construct, we also performed RNA purification by centrifuging 16 × 10^6^ cells, washing them with 4 ml PBS, splitting into four tubes of 1 ml, and purifying RNA using NucleoSpin RNA II kit (MACHEREY-NAGEL) according to the manufacturer's protocol. This purification was also performed in two replicates.

For the transient transfection experiments, each of the replicates was harvested for RNA purification, washed with PBS, and split into tubes not exceeding 5 × 10^6^ cells per tube. The tubes were flash frozen in liquid nitrogen and RNA was purified using NucleoSpin RNA II kit (MACHEREY-NAGEL) according to the manufacturer's protocol. The purified RNA was treated with DNAse (QIAGEN) in solution according to the manufacturer's protocol, and purified again using NucleoSpin RNA II kit (MACHEREY-NAGEL).

Preparing samples for sequencing: in order to maintain the complexity of the library amplified from gDNA, PCR reactions were carried out on a gDNA amount calculated to contain a minimum average of 200 copies of each oligo included in the sample. For each of the 16 bins, we used 20 µg of gDNA as template in a two-step nested PCR performed in two tubes (to include the required amount of gDNA), each containing 100 μl (in both steps). In the first step, each reaction contained 10 μg gDNA, 50 μl of Kapa Hifi ready mix X2 (KAPA Biosystems), 5 μl of 10 μM 5′ primer, and 5 μl of 10 μM 3′ primer. The parameters for the first PCR were 95 °C for 5 min, 18 cycles of 94 °C for 30 s, 61 °C for 30 s, and 72 °C for 30 s, each, and one cycle of 72 °C for 5 min. The primers used for this reaction were CACTCCAAGACCGAGCTCAACTTC (Fw primer) and GGGGCTTTTCTGTCACCAATCC (Rv primer). Multiple PCR reaction products of each bin were combined. In the second PCR step, each reaction contained 5 μl of the first PCR product (uncleaned), 50 μl of Kapa Hifi ready mix X2 (KAPA Biosystems), 5 μl of 10 μM 5′ primer, and 5 μl of 10 μM 3′ primer. The PCR program was 95 °C for 5 min, 24 cycles of 94 °C for 30 s, 65 °C for 30 s, and 72 °C for 30 s, each, and one cycle of 72 °C for 5 min. Specific primers corresponding to the constant region of the plasmid were used. The 5′ primer also had a unique upstream 8 nt barcode sequence and another 5 nt random sequence (underlined) (5′-HNHNHXXXXXXXX CGAAATGGGCCGCATTGC-3′, where “X”s represent barcode nucleotides, “N”s represent random nucleotides and “H”s represent any nucleotide except “G”), and three different barcodes were used for each bin. The 3′ primer was common to all bins (5′-HNHNHNHNTCGTCATCAGCCGCAGTG-3′). Multiple PCR reaction products of each bin were combined. The concentration of the PCR samples was measured using a Quant-iT High-Sensitivity dsDNA Assay Kit (Invitrogen) in a monochromator (Tecan i-control), and the samples were mixed in ratios corresponding to their ratio in the population, as defined when sorting the cells into the 16 bins. The library was separated from unspecific fragments by electrophoresis on a 1.5% agarose gel stained by EtBr, cut from the gel, and cleaned in 2 steps: gel extraction kit (Qiagen) and SPRI beads (Agencourt AMPure XP). The sample was assessed for size and purity at the Tapestation, using high-sensitivity D1K screenTape (Agilent Technologies, Santa Clara, California). We used 10 ng library DNA for library preparation for NGS; specific Illumina adaptors were added, and DNA was amplified using 14 amplification cycles, protocol adopted from Blecher-Gonen et al.^67^. The sample was reanalyzed using Tapestation.

For the mCherry single integration population, we performed the two-step nested PCR on both gDNA and cDNA prepared from the same population of cells. Since the reverse primer used in the first PCR on the gDNA from the 16 bins anneals downstream to the polyadenylation signal, and since we required the same PCR reaction setup and program on the gDNA and cDNA of the mCherry single integration population, we used a primer that anneals between the library insertion site and the polyadenylation signal. The primer sequence used was CCCTCACTAAAGGGAAAGGGTCC. We prepared cDNA in a single reverse transcription reaction for each replicate using SuperScript® III First-Strand Synthesis System (ThermoFisher Scientific) with random hexamer primers and 5 μg of input RNA, according to the manufacturer's protocol. The cDNA product was diluted 1:10 and 2 μl were used as a template in each of ten reactions of the first PCR step. The PCR reaction setup and program were the same as used for the first PCR step on the gDNA from the 16 bins. PCR products from the ten reactions were pooled and 5 μl were used as the template for each of two reactions of the second PCR step performed for each replicate. For the gDNA from the same population, we used 40 µg of gDNA as template in four tubes (to include the required amount of gDNA). We used the same primers, PCR reaction setup and program, as we used for the PCR steps on the cDNA. We used 50 ng library DNA for library preparation for NGS with specific Illumina adaptors and DNA was amplified using 14 amplification cycles. The samples were analyzed using Tapestation.

For RNA purified from the transient transfection experiments, we prepared cDNA in a two reverse transcription reactions for each replicate using SuperScript® III First-Strand Synthesis System (Thermo Fisher Scientific) with random hexamer primers and 2.5 μg of input RNA per reaction, according to the manufacturer's protocol. The cDNA products were pooled together and 2 μl were used as template for each of four amplification reactions. Each amplification reaction contained 2 μl of the cDNA template, 25 μl of Kapa Hifi ready mix X2 (KAPA Biosystems), 2.5 μl of 10 μM 5′ primer, 2.5 μl of 10 μM 3′ primer, and 18 μl of ddw. The PCR program was 95 °C for 5 min, 20 cycles of 98 °C for 20 s, 65 °C for 15 s, and 72 °C for 30 s, each, and one cycle of 72 °C for 5 min. The primers used for this reaction were CGAAATGGGCCGCATTGC (Fw primer) and TCGTCATCAGCCGCAGTG (Rv primer).

Amplification of the library from the plasmid DNA used for the transient transfection was also performed with Kapa Hifi ready mix (KAPA Biosystems). A single amplification reaction included 1 μl of the cDNA template, 25 μl of Kapa Hifi ready mix X2 (KAPA Biosystems), 2.5 μl of 10 μM 5′ primer, 2.5 μl of 10 μM 3′ primer, and 19 μl of ddw. The PCR program was 95 °C for 5 min, 14 cycles of 98 °C for 20 s, 65 °C for 15 s, and 72 °C for 30 s, each, and one cycle of 72 °C for 5 min. The primers used were the same ones used for the amplification of cDNA.

The library amplification products from the RNA and plasmid DNA were each separated from unspecific fragments by electrophoresis on a 1.5% agarose gel stained by GelStar (Cambrex Bio Science Rockland), cut from the gel, and cleaned in 2 steps: gel extraction kit (Qiagen) and SPRI beads (Agencourt AMPure XP). Each sample was assessed for size and purity at the Tapestation, using high-sensitivity D1K screenTape (Agilent Technologies, Santa Clara, California). For each sample 20 ng library DNA were used for library preparation for NGS; specific Illumina adaptors were added, and DNA was amplified using 14 amplification cycles, protocol adopted from Blecher-Gonen et al.^67^. The sample was reanalyzed using Tapestation.

### Computational analyses

Mapping next generation sequencing reads: to determine the identity of the oligo after sequencing, a unique 12-mer barcode sequence was placed at the 5′ end of each variable region. Barcodes were designed to differ from other barcodes in the library by 3 nt or more and to avoid the introduction of known regulatory elements such as seed regions for highly expressed miRNAs, the canonical polyadenylation signal (AATAAA), and its point mutants. DNA was sequenced on NextSeq-500 sequencer. For the 16 bins, we obtained a total of ~60 million paired-end reads. For the gDNA of the mCherry single integration population, we obtained ~58 and ~46 million reads for replicates 1 and 2, respectively. For the cDNA of the mCherry single integration population, we obtained ~74 and ~43 million reads for replicates 1 and 2, respectively. For the cDNA from the transient expression experiment, we obtained 8–12 million reads per sample. For the plasmid DNA, we obtained ~11 million reads. For the 16 bins, as reference sequence for mapping, we constructed in silico an “artificial genome”. Each chromosome in the genome corresponds to a specific bin barcode (total 48 chromosomes). Each chromosome is composed of repeats of the 8 nt bin barcode, 18 nt constant region, 12 nt variant barcode, 5 nt from the variable region (43 nt total), and 60 “N”s. Paired-end NextSeq reads in the length of 75 nt were trimmed to 50 nt containing the 5 nt random sequence, bin barcode, common priming site, and the variant barcode. Trimmed reads were mapped to the artificial library genome using Novoalign aligner, filtered for minimal mapping quality of 60, and for perfectly aligned reads for the entire 43 nt region, and the number of reads for each designed oligo was counted in each bin. For the mCherry single integration population gDNA and cDNA, as reference sequence for mapping, we constructed a single “artificial chromosome” composed of repeats of the 18 nt constant region, 12 nt variant barcode, 5 nt from the variable region (35 nt total) and 60 “N”s. Single-end Next seq reads in the length of 75 nt were trimmed to 43 nt containing the common priming site and the variant barcode. The trimmed reads were mapped to the artificial library chromosome using Novoalign aligner, filtered for minimal mapping quality of 60 and for perfectly aligned reads for the entire 35 nt region and the number of reads for each designed oligo was counted in each sample. Analysis of the cDNA from the transient transfection experiment and the plasmid DNA was performed similarly to the analysis of the gDNA and cDNA from the mCherry single integration population.

Computing protein repression scores: for the 16 bins, we arranged the data in a matrix of read counts, where each row corresponded to a variant in our library (14,151 rows) and each column corresponded to a bin barcode (48 column). The columns were normalized by dividing each cell by the sum of reads in that column and then multiplying by the proportion of cells sorted into that bin. The data from the three barcodes for each bin was summed per bin to produce a matrix of normalized reads in which each of 16 columns corresponded to data from one bin. To this matrix, we applied a number of filters to reduce experimental noise. First, for variants with a row sum less than 100, all cells were set to zero. Second, for cells with a value less than five, the cell value was set to zero. Third, we observed cases in which the calculated expression levels were underestimated (as judged by eye examination of the distribution across 16 bins) due to a disproportionally large number of reads in bins one and two. Therefore, we zeroed bins one and two when they both had a normalized read count higher than 50 and the read count in bin three was <20% than the count in either bin one or two, as they were unlikely to be part of the correct peak corresponding to higher expression. Fourth, we zeroed all the cells of variants that had >70% of the normalized reads in bins one and two, since given the technical noise in those bins, we were not able to reliably calculate the expression levels for these cases. Fifth, for each variant, we set to zero cells surrounded by zero values (isolated cells). Sixth, for each variant, we set all cells to zero if the sum of normalized reads after filtering was less than 60% of the sum of normalized reads before filtering. Finally, we zeroed values for all cells of variants that had more than one peak. For each variant, we normalized the vector of values across the 16 bins so it sums to one and applied a Savitzky–Golay filter for smoothing the data. We detected peaks in the smoothed vector by simple approach in which a point is considered a maximum peak if it has the maximal value, and was preceded (to the left) by a value lower by delta (which we set to 0.05).

For each bin, we calculated the $\mathrm{median}\left( {{{\mathrm{log}}_2}\left( {\frac{{mNeonGreen}}{{mCherry}}} \right)} \right)$ as a measure of the bin center based on recorded FACS data. For each variant, we computed the mean expression as the weighted average of bin centers, where the weight of each bin is the fraction of the normalized reads number in this bin of its total normalized reads in all 16 bins. Thus, the expression values are in log2 scale. We calculated the $\mathrm{median}\left( {{ log_2}\left( {\frac{{mNeonGreen}}{{mCherry}}} \right)} \right)$ for the vector with no insert was 5.24, as calculated from recorded FACS analysis data. To calculate protein repression values, we subtracted the mean expression values from the expression value of the vector with no insert. These values were used throughout the analysis. For variants with no repression value, we set the value to None.

Computing normalized RNA levels: we arranged the read counts from the mCherry single integration population gDNA and cDNA data in a matrix, where each row corresponded to a variant in our library (14,151 rows) and each column corresponded to one of the two replicates of either the gDNA or cDNA samples (four columns). To compare between replicates, we calculated for each variant that had more than 100 reads in all four samples the ${log_2}\left( {\frac{{\mathrm{cDNA}\;\mathrm{reads}}}{{\mathrm{gDNA}\;\mathrm{reads}}}} \right)$ as an estimate for normalized RNA levels. Since the agreement between the replicates was high (*R* = 0.82, *p* < 10^−10^), we summed the reads between replicates for each variant. For variants that had more than 100 reads for both the combined cDNA and combined gDNA samples, we calculated the ${ log_2}\left( {\frac{{\mathrm{cDNA}\;\mathrm{reads}}}{{\mathrm{gDNA}\;\mathrm{reads}}}} \right)$. For the rest of the variants, the normalized RNA level was set to None.

For the transient transfection experiment, we analyzed the read counts of each variant in our library for the two replicates for each of the four cell lines. To compare between replicates, we calculated for each variant the ${log_2}\left( {\frac{{\mathrm{cDNA}\;\mathrm{reads}}}{{\mathrm{plasmid}\;\mathrm{DNA}\;\mathrm{reads}}}} \right)$ as an estimate for normalized RNA levels. Since the agreement between the replicates was high (*R* = 0.95–0.97, *p* < 10^−10^, depending on the cell line), we summed the reads between replicates for each variant. For variants that had more than 10 reads for the combined cDNA samples, we calculated the ${log_2}\left( {\frac{{\mathrm{cDNA}\;\mathrm{reads}}}{{\mathrm{plasmid}\;\mathrm{DNA}\;\mathrm{reads}}}} \right)$. For the rest of the variants, the normalized RNA level was set to None. In order to compare the reporter expression levels between the cell lines, we applied a Z score transformation to the normalized RNA levels.

Calculation of ΔΔ*G*: we scored miRNA-target interactions by an energy score, ΔΔ*G*, equal to the difference between the energy gained by binding of the miRNA to the target, Δ*G*_hybridization_, and the energy required to make the target region accessible for miRNA binding, Δ*G*_open_. Δ*G*_hybridization_ is the binding free energy of the miRNA-target duplex structure in which the miRNA and target are paired according to pairing constraints imposed by the seed. To compute this value, we used RNAhybrid version 2.1.2^68^ with the following parameters: energy threshold −0.01, helix constraint from 2 to 7, max internal loop size of five, max bulge loop size of five, the 210 nt long variant sequence, and the miRNA sequence. When analyzing the effect of the MRE accessibility of a given site (as in Fig. 4b, c), in case of multiple hits the data for the analyzed MRE was extracted from the RNAhybrid results based on the position within the oligo. The code returned the predicted hybridization energy (Δ*G*_hybridization_) and the predicted hybridization structure from which we extracted the start and end position of the binding site. To predict Δ*G*_open_ we used the pf_fold_par function from the ViennaRNA 2.0^60^ python wrapper, which returns the Gibbs free energy of the ensemble and can have a structure constraint as input. We used the start and end positions computed from the RNAhybrid results to compute this structure constraint. The input sequence used was the 210 nt long variant sequence flanked with additional 70 nucleotides from the vector upstream and downstream. This ensured that there were at least 70 nucleotides surrounding the interaction site, since this reduces the time complexity of the calculation and there is low probability of secondary structure base-pairing interactions between nucleotides that are separated by more than 70nt^18^. We then calculated Δ*G*_open_ as the difference between the Gibbs free energy of the ensemble without constraints and the Gibbs free energy of the ensemble with the structure constraint. Finally, we computed ΔΔ*G* with the formula ΔΔ*G* = Δ*G*_hybridization_ – Δ*G*_open_.

Feature extraction for prediction model: for feature extraction, we considered 274 miRNA sequences expressed in K562 cells with a mean expression above a threshold of 0.1 according to a published microarray data set^34^. For each miRNA-variant pair, we computed ΔΔ*G* for each of multiple hits returned by RNAhybrid with the constraint of seed pairing at positions 2–7. To integrate multiple sites with ΔΔ*G* scores for one miRNA on the same variant, we compute an overall miRNA-variant interaction score according to ${\it{\Delta \Delta }}G_T = - ln{\sum} {e^{ - \Delta \Delta G}}$^18^. If the miRNA has no hits in the target sequence, an arbitrarily high value of 50 kcal/mol is used. This score is the ΔΔ*G* used for further analysis.

Prediction of protein repression and normalized RNA levels: the data used for our model consisted of 12,339 variants, for which we quantified both protein repression levels and mRNA expression levels. The data excluded the subset of variants, which varied only in the barcode sequence. We used ~90% of the data for the model development phase and ~10% for the final model validation.

All predictions were made using stochastic GBR. Our predictor was based on the code adapted from the sklearn 0.17.1 GradientBoostingRegressor class. We performed a parameter optimization step based on a random selection of 50 parameter sets. We optimized alpha, learning rate, max depth, max features, and n_estimators. To select the best parameter set, we generated 10-fold cross validation predicted values and computed the Pearson correlation coefficient between the predicted and measured values as an estimate of model performance. The parameter set with the highest performance was selected and used in the final predictor. To assess the performance of our model on training data, we employed an outer 10-fold cross validation scheme. We divided the training data into ten subsamples, applied our model on nine of the subsamples and predicted the values of the tenth subsample. These cross validation predicted values were then plotted against the measured values (as in Figs. 5a and 6c) and the Pearson correlation between them was computed. To assess the performance of our model on the held out test data, we trained the model on all of our training data, predicted the values for the held out test data, and assessed the performance using Pearson correlation.

To assess model performance with RNA level as the only feature input to the model, we applied the same scheme described for assessing the performance on training data, but used the RNA level as the only input feature. To assess model performance with 274 MRE-based features and RNA level, we applied the same scheme, but added the RNA level as an additional input feature to the MRE-based features.

For the elastic net (EN) model, we used the same framework as for GBR. For parameter optimization, we optimized the values of alpha and l1_ratio. We trained the model on our training data and tested its performance on the held out data. For the basic model, the parameter optimization was not required. Thus, we trained a linear regression model on our training data for each of the miRNAs, predicted on our held out data values using each model and averaged these values for the final prediction outcome. Such a model does not allow for learning of any interactions between the miRNAs.

Prediction of paired difference in repression for WT sequences: using the variants that had protein repression measurements, we constructed a data set composed of all WT and mutant pairs in the WTs set. The data contain 929 WT and mutant pairs spanning 472 unique WT sequences. For each pair, the difference in fold repression was calculated by subtracting the fold repression of the WT from the fold repression of the mutant. For each pair, we concatenated the WT and mutant features to form a vector of features used for that pair. Since we observed some outliers in the data, we applied an outlier detection scheme in which variants differing more than five times the median absolute deviation from the median paired difference in repression were filtered (total of three pairs). We randomly sampled ~10% of the data and kept it untouched until finishing model development. The rest of the prediction pipeline was the same as the one used for predicting protein repression.

### Statistical analysis

Statistical analysis was performed using the scipy 0.17 python package. When comparing two groups, the Mann–Whitney *U* test was used. When FDR correction was applied, the Benjamini–Hochberg procedure was used as implemented in the mne 0.12 python package

### Code availability

The code used in this study is available from the corresponding author upon reasonable request. For data analysis, we used python 2.7.11 with pandas 0.18, seaborn 0.6, scipy 0.17, sklearn 0.17.1, and mne 0.12.

### Data availability

The data that support the findings of this study are available from the corresponding author upon reasonable request. Sequencing data can be accessed with SRA study ID SRP128656.

## Electronic supplementary material

Supplementary information
