# Supplementary material for: Unraveling the determinants of microRNA mediated regulation using a massively parallel reporter assay
Source: Nat Commun. 2018 Feb 6;9:529. doi: 10.1038/s41467-018-02980-z (PMC5802814; doi:10.1038/s41467-018-02980-z)
Supplement: Supplementary file 1 — Supplementary information [file 41467_2018_2980_MOESM1_ESM.pdf]

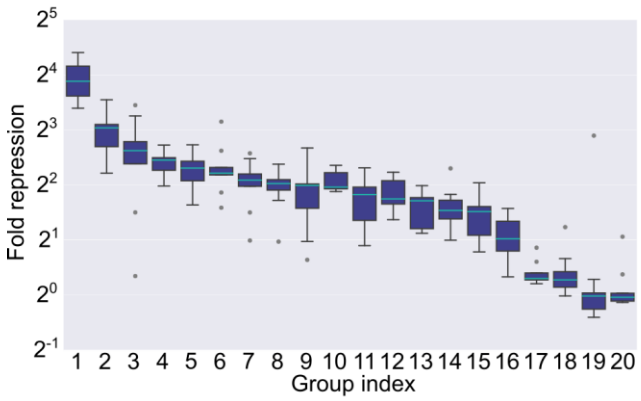

Supplementary Figure 1 – Effect of barcode variability on protein repression. Comparison of protein repression distribution across groups of variants. Each group contains up to ten variants differing only in the barcode sequence. Variants span a wide protein repression range and show low median RSD (10.5%), estimated across all groups.

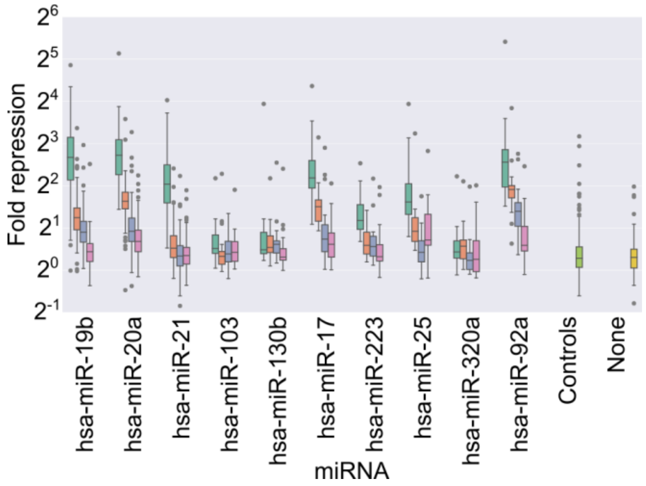

Supplementary Figure 2 – Effect of MRE type on protein repression per miRNA. Designed MREs of a given type for the selected ten miRNAs were placed in up to 57 contexts at a single copy in either of two positions to generate a diverse set of variants. The boxplots present a comparison of fold repression for variants grouped by the MRE type for each miRNA.

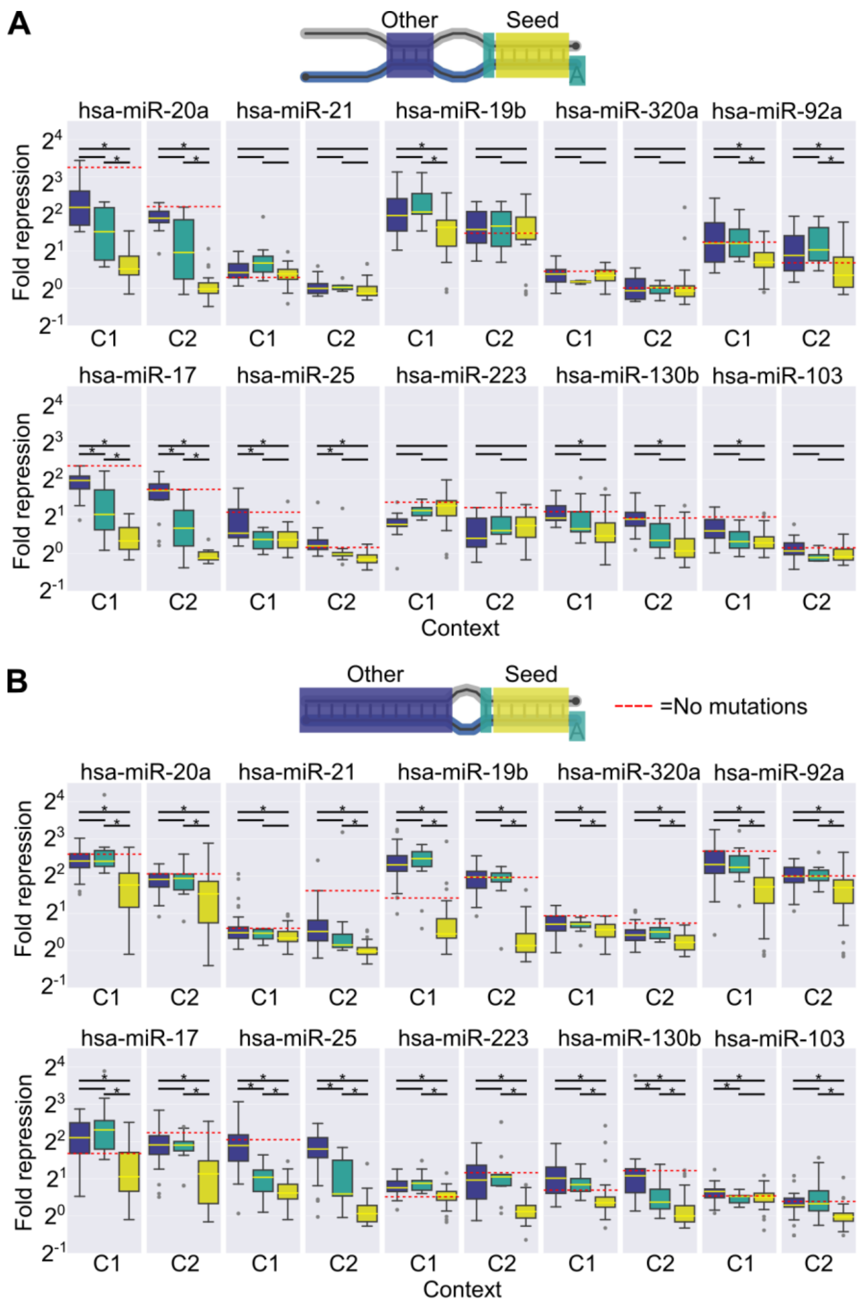

Supplementary Figure 3 – Effect of MRE mutations region on protein repression. Comparison of the effect on fold repression of mutations in three regions of the MRE for the ten miRNAs for 3' complementary (A) and bulged (B) MRE types. The first position is considered to be mutated if it is not an 'A'. The effect of mutations in the different regions varies between miRNAs. Boxplot pairs marked with * are significantly different ($p<0.05$, Mann-Whitney U one sided test, FDR corrected for multiple testing). The fold repression level of the non-mutated MRE in each context is indicated. In each variant two identical copies of the MREs were placed in the context in order to increase sensitivity.

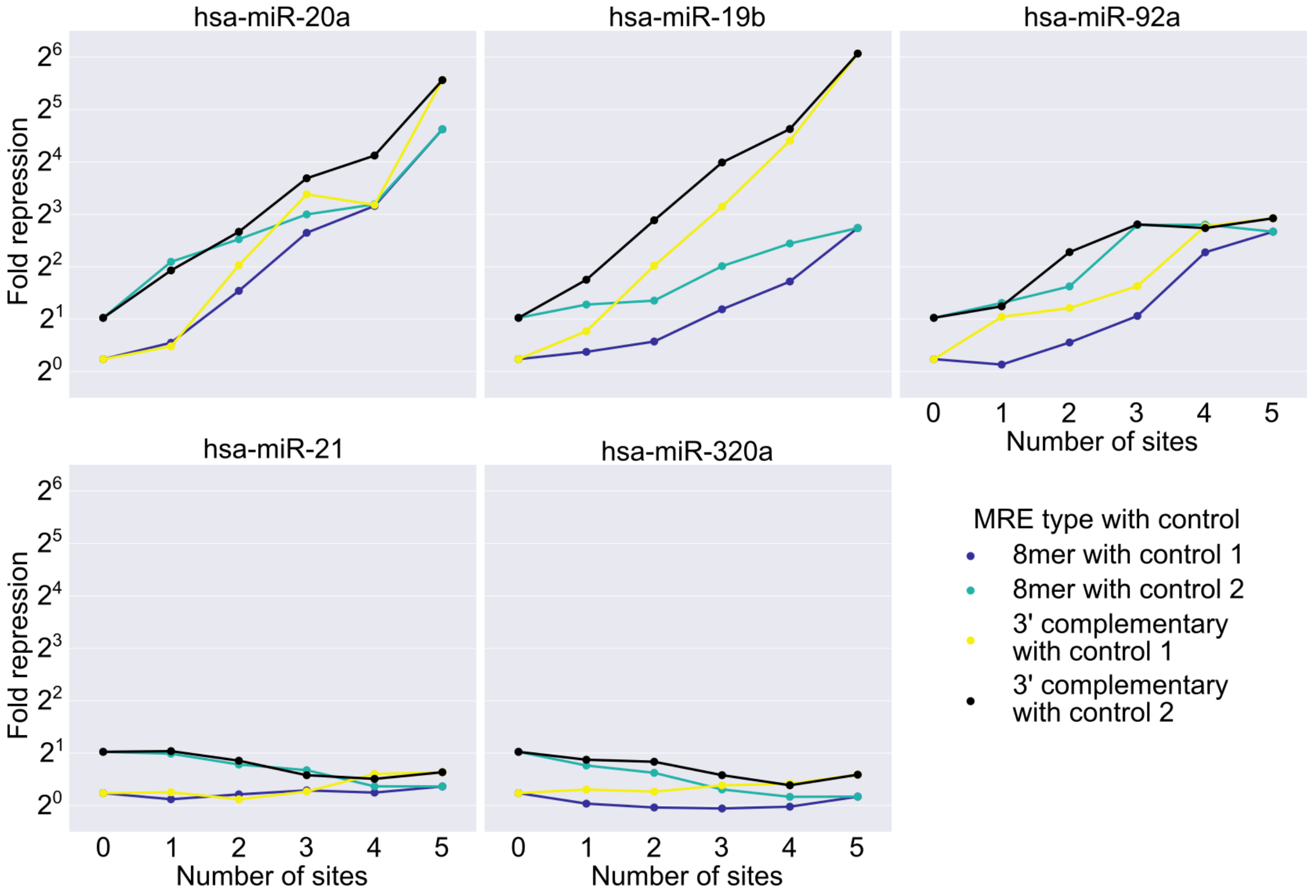

Supplementary Figure 4 – Effect of MRE multiplicity on protein repression. For each MRE and control pair, the variants were aggregated by the number of MRE sites and their median fold repression was plotted as a function of the number of sites.

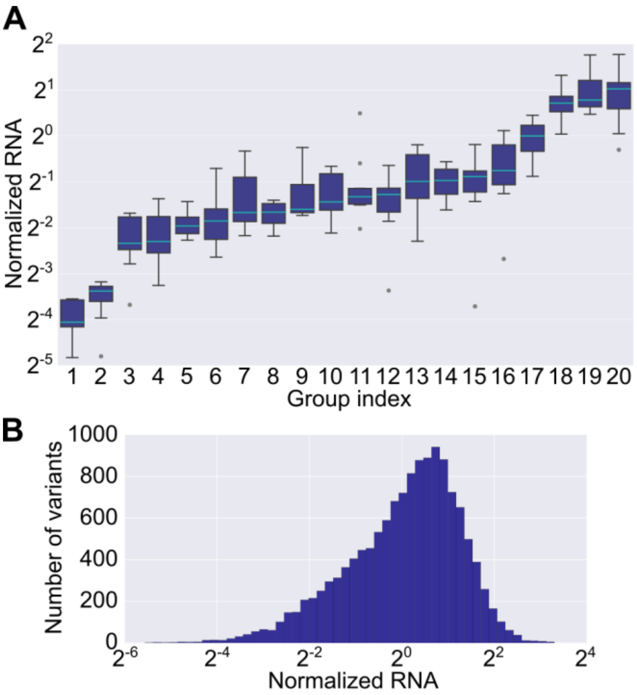

Supplementary Figure 5 - The normalized RNA measurements are more susceptible to technical noise. (A) Comparison of normalized RNA distribution across groups of variants. Each group contains up to ten variants differing only in the barcode sequence. Variants span a wide normalized RNA range and have a median RSD of 34%, estimated across all groups. (B) A histogram depicting the distribution of normalized RNA levels computed from the combined data of the two replicates.

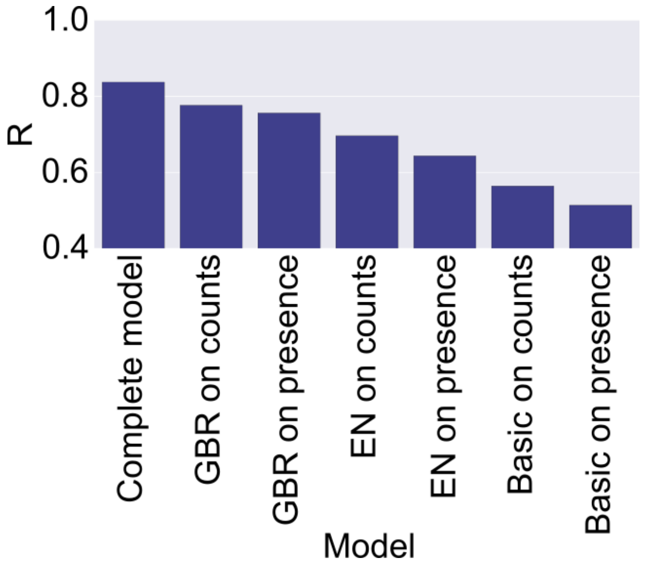

Supplementary Figure 6 – Simpler computational models reveal contributing factors to model performance. We devised a number of simpler models. First, we used an elastic net (EN) model on the seed counts features, thus constructing a regularized linear regression model for all the miRNAs combined. Second, we applied our GBR model on the seed counts features, facilitating learning of non-linear interactions between features. Third, we devised a basic model which uses linear regression based prediction for each feature separately and averages the predicted values. Finally, we applied each of the three models considering only the presence or absence of binding sites for each miRNA. The bar plot presents the performance of each model on held out data. Performance is quantified as the pearson correlation between predicted and measured values. The models were described in the main text.

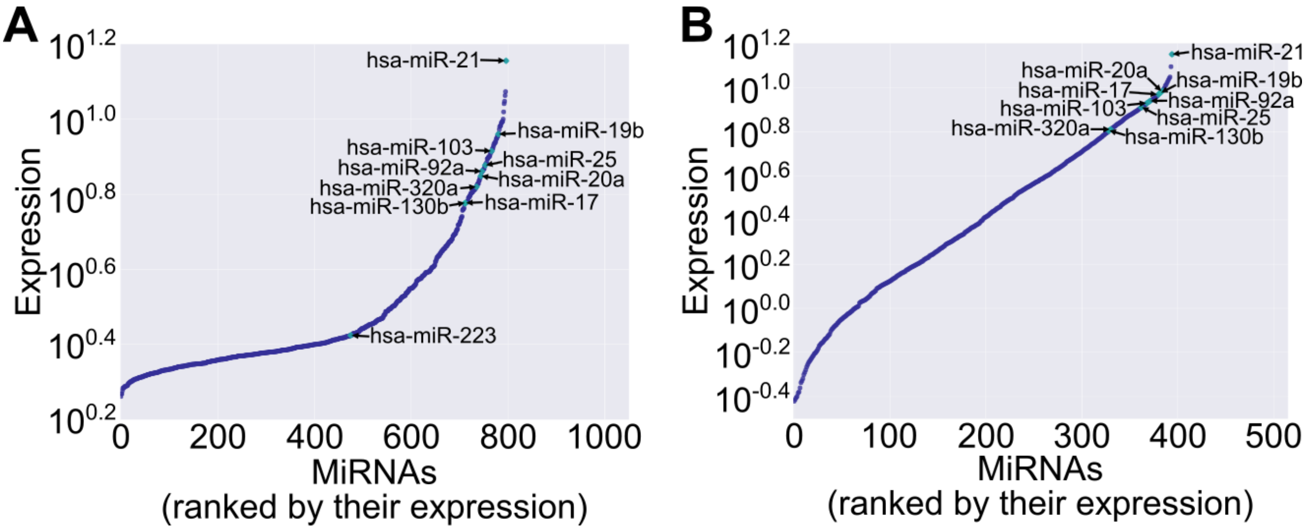

Supplementary Figure 7 – Expression levels of miRNAs in MCF7 and HepG2. MiRNAs expressed in MCF7 (A) and HepG2 (B) cells as quantified by a published microarray experiments[1,2]. The ten highly expressed miRNAs selected for the library design are indicated. In HepG2 hsa-miR-223 was below the detection limit.

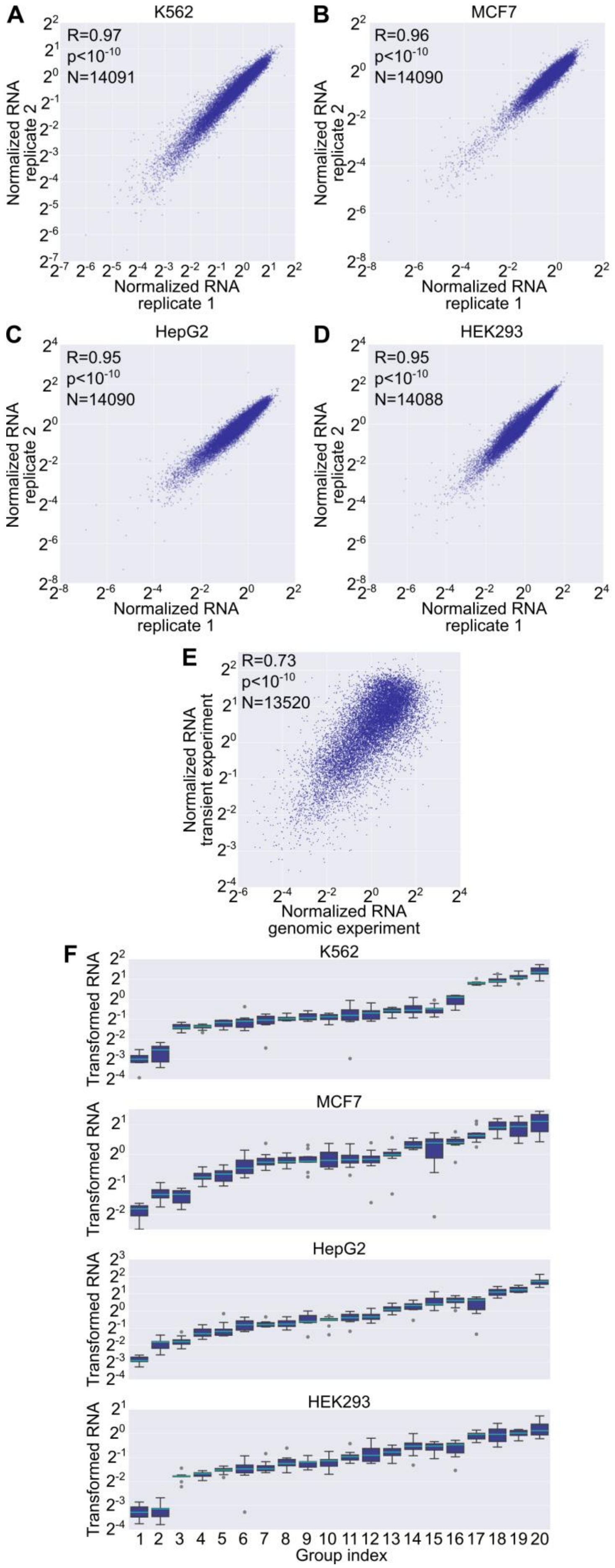

A
K562
R=0.97
p<10^-10
N=14091
Normalized RNA replicate 2
Normalized RNA replicate 1
B
MCF7
R=0.96
p<10^-10
N=14090
Normalized RNA replicate 2
Normalized RNA replicate 1
C
HepG2
R=0.95
p<10^-10
N=14090
Normalized RNA replicate 2
Normalized RNA replicate 1
D
HEK293
R=0.95
p<10^-10
N=14088
Normalized RNA replicate 2
Normalized RNA replicate 1
E
R=0.73
p<10^-10
N=13520
Normalized RNA transient experiment
Normalized RNA genomic experiment
F
K562
Transformed RNA
MCF7
Transformed RNA
HepG2
Transformed RNA
HEK293
Transformed RNA
1 2 3 4 5 6 7 8 9 10 11 12 13 14 15 16 17 18 19 20
Group index

Supplementary Figure 8 – RNA level measurements from transient transfection are reproducible and less susceptible to noise. (A-D) Scatter plot of normalized RNA level measurement replicates in K562, MCF7, HepG2 and HEK293 cells respectively. (E) Scatter plot of normalized RNA level measurements from the transiently transfected reporter library vs the genomically integrated one. The differences in the measurements can be attributed to the differences in experimental procedures. (F) Comparison of z-score transformed RNA expression distribution across groups of variants for K562, MCF7, HepG2 and HEK293. Each group contains up to ten variants differing only in the barcode sequence. The groups are numbered by their ranked median expression levels and the sequences in each group differ between cell lines. The estimated median RSD, estimated across all groups, is 19%, 22%, 18% and 25% for K562, MCF7, HepG2 and HEK293 respectively.

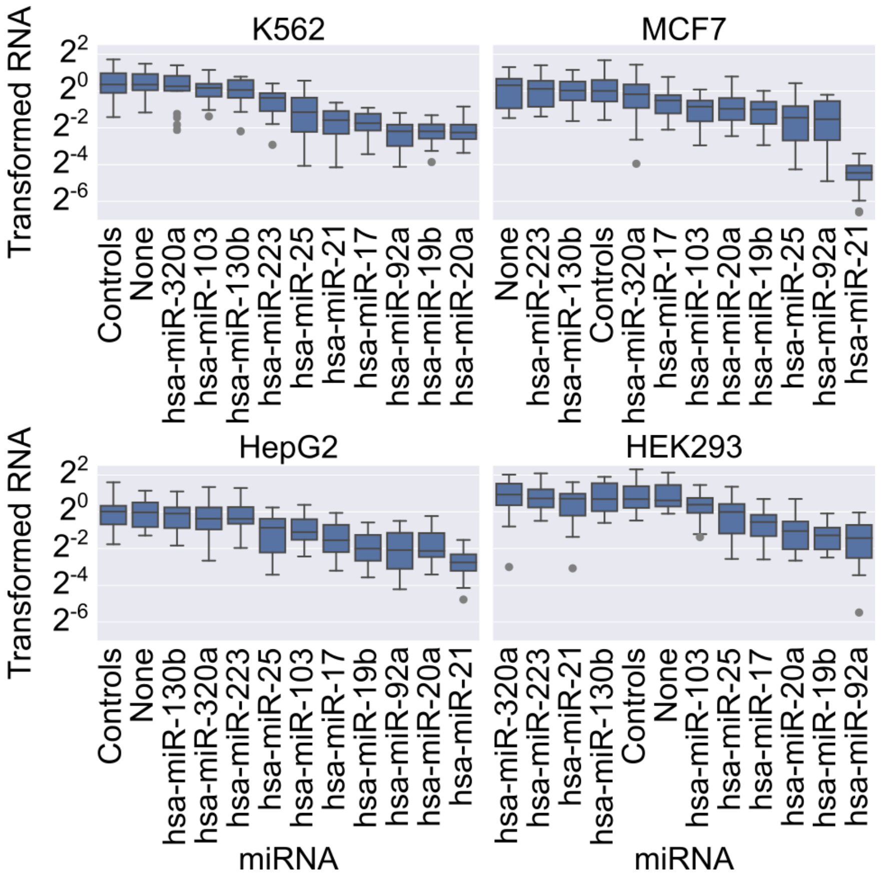

Supplementary Figure 9 – Ranking of miRNAs by their activity differs between cell lines. Comparison of transformed RNA levels as a function of different miRNA identities for K562, MCF7, HepG2 and HEK293 cells. Each boxplot contains up to 20 variants generated by placing MREs, perfectly matched to their miRNAs, in varying contexts in two positions. The ranking of the miRNAs by the median reporter repression differs between the cell lines.

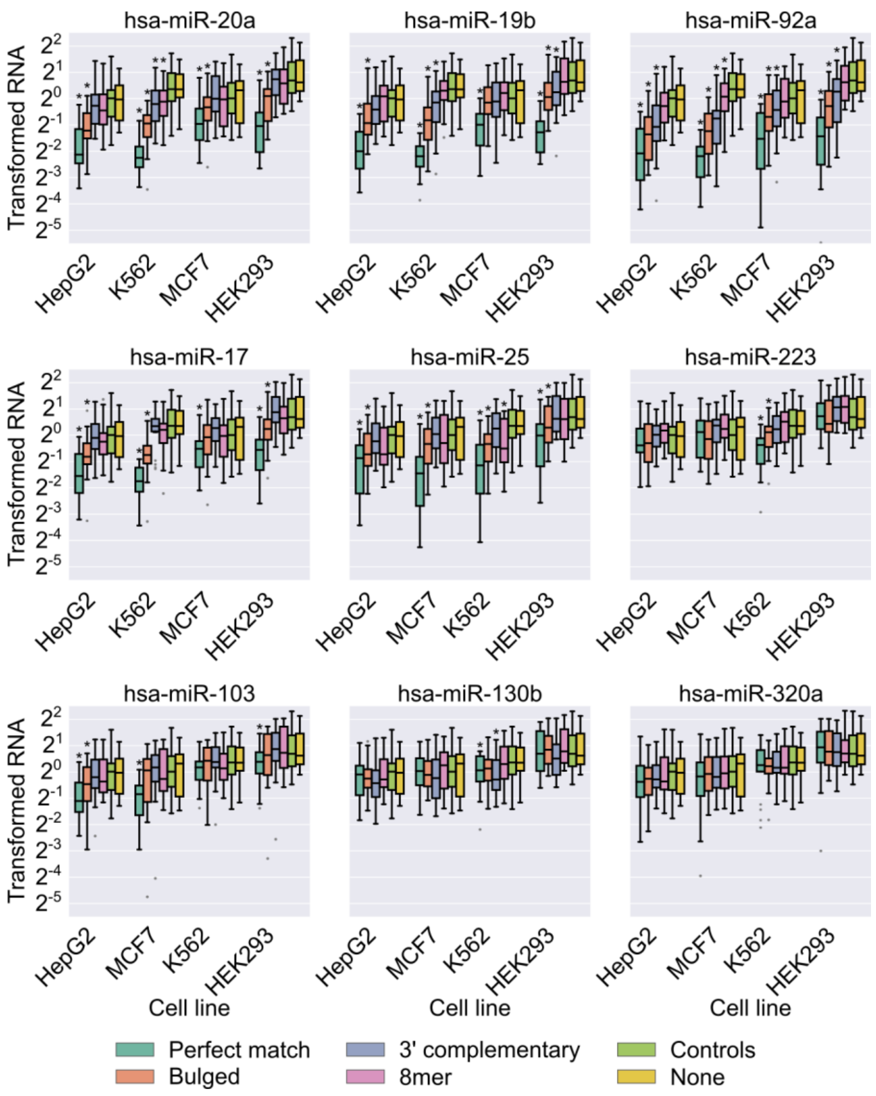

Supplementary Figure 10 – The effect of the MRE type is consistent across miRNAs and cell lines. Transformed RNA levels of reporter constructs with different MRE types for the indicated miRNAs in different cell lines. Controls contain control sequences instead of the MRE. None group contains the context with no inserted sequences. Boxplots marked with * are significantly different ($p<0.05$, Mann-Whitney U one sided test) from the controls group.

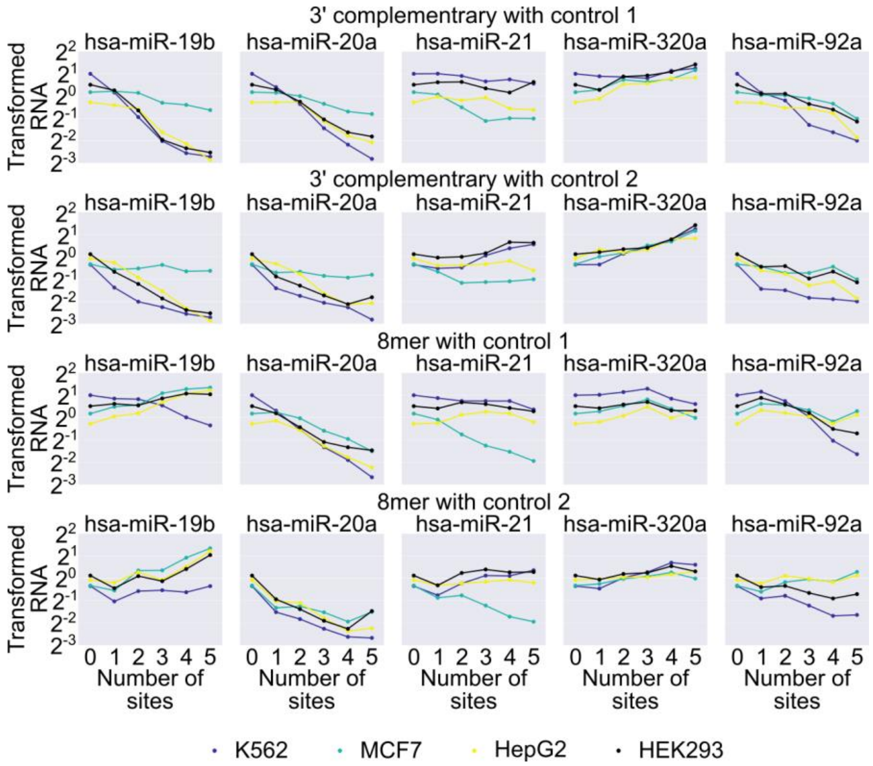

Supplementary Figure 11 – Effect of MRE multiplicity on RNA levels across cell lines. Transformed RNA levels as a function of number of MRE sites for K562, MCF7, HepG2 and HEK293 cells. Analysis performed as in Figure 4E per cell line. Colors represent results from different cell lines as shown in the legend.

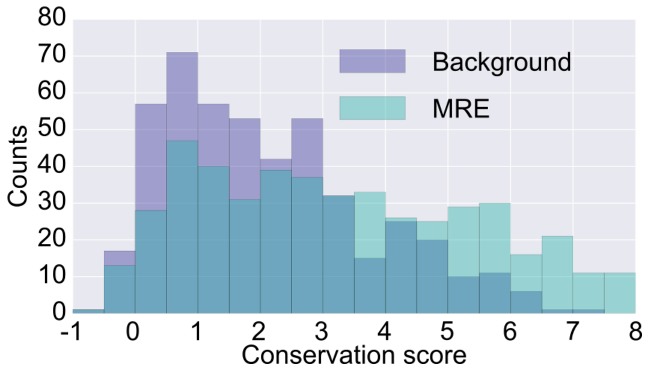

Supplementary Figure 12 – The MRE sequence is more conserved than background sequences in the analyzed WT variants. Conservation scores (phyloP100way) for the entire variable region for each of our WT variants were extracted from the UCSC genome browser. To assess the conservation level of the MREs, a 12bp window was chosen at the MRE 3' end (positions 1-12). Shown is a histogram of the average conservation scores of the MREs along with a histogram of the average conservation scores of the rest of the native sequences. The results show that the MREs are significantly more conserved than the rest of the WT sequences ($p<10^{-10}$, Wilcoxon signed-rank test).

References

1. Camps, C. *et al.* Integrated analysis of microRNA and mRNA expression and association with HIF binding reveals the complexity of microRNA expression regulation under hypoxia. *Mol. Cancer* **13,** 28 (2014).
2. He, X.-X. *et al.* MicroRNA-375 targets AEG-1 in hepatocellular carcinoma and suppresses liver cancer cell growth in vitro and in vivo. *Oncogene* **31,** 3357–3369 (2012).
